# On-Site Validation of a Microwave Breast Imaging System, before First Patient Study

**DOI:** 10.3390/diagnostics8030053

**Published:** 2018-08-18

**Authors:** Angie Fasoula, Luc Duchesne, Julio Daniel Gil Cano, Peter Lawrence, Guillaume Robin, Jean-Gael Bernard

**Affiliations:** MVG Industries, 91140 Villebon sur Yvette, France; luc.duchesne@mvg-world.com (L.D.); julio_daniel.gil_cano@mvg-world.com (J.D.G.C.); peter.lawrence@mvg-world.com (P.L.); guillaume.robin@mvg-world.com (G.R.); jean-gael.bernard@mvg-world.com (J.-G.B.)

**Keywords:** breast cancer diagnosis, microwave imaging, medical radar, on-site validation, breast phantoms

## Abstract

This paper presents the Wavelia microwave breast imaging system that has been recently installed at the Galway University Hospital, Ireland, for a first-in-human pilot clinical test. Microwave breast imaging has been extensively investigated over the last two decades as an alternative imaging modality that could potentially bring complementary information to state-of-the-art modalities such as X-ray mammography. Following an overview of the main working principles of this technology, the Wavelia imaging system architecture is presented, as are the radar signal processing algorithms that are used in forming the microwave images in which small tumors could be detectable for disease diagnosis. The methodology and specific quality metrics that have been developed to properly evaluate and validate the performance of the imaging system using complex breast phantoms that are scanned at controlled measurement conditions are also presented in the paper. Indicative results from the application of this methodology to the on-site validation of the imaging system after its installation at the hospital for pilot clinical testing are thoroughly presented and discussed. Given that the imaging system is still at the prototype level of development, a rigorous quality assessment and system validation at nominal operating conditions is very important in order to ensure high-quality clinical data collection.

## 1. Introduction

Microwave imaging for medical applications has been of interest for many years. The microwave images are maps of the electrical property distributions in the body. The electrical properties of various tissues may be related to their physiological state; notably, there has been some evidence of changes in the properties of cancerous tissues when compared to normal tissues. Cancer detection with microwave imaging is based on this contrast in electrical properties. Microwave imaging, as an alternative imaging modality to X-ray mammography for breast cancer detection, has interested many researchers during the last 20 years [1,2,3,4,5].

Among them, at least four research teams have performed clinical testing of their experimental prototypes [6,7,8,9,10,11], demonstrating numerous positive results and a potential added value of the microwave technology toward a better specificity and/or sensitivity in breast cancer diagnosis when combined with the state-of-the-art modalities. The potential for regular follow-up of the patient during breast cancer treatment has also been envisaged using the microwave technology [8]. The interested reader is directed to a series of review papers that have been recently published [12,13,14]; these papers provide an extensive overview of the microwave breast imaging system prototypes that have been clinically tested, the principal technical features and differences among them, as well as the most important results reported so far.

One of the most appealing features of the microwave technology is the use of non-ionization radiation; thus, it is very safe for the patient and could open up the possibility for scheduling regular three-dimensional (3D) scans of the breast, as often as required, for optimal diagnostic and/or follow-up of rapidly evolving pathologies. In conjunction with the design of appropriate radar signal processing algorithms, automated tumor detection can be naturally integrated with the microwave image formation process, providing clinicians with useful tools for computer-aided diagnosis (CAD). Several CAD systems for breast cancer have been proposed during the last decade [15,16,17], especially on X-ray mammography systems. Although they are not yet part of the routine clinical practice, they have proved useful in aiding clinicians to diagnose breast cancers in cases where simple visual inspection is ambiguous. The Wavelia microwave breast imaging system, as presented in this paper, is being developed for such an intended future use.

The Wavelia system is a low-power electromagnetic wave breast imaging device for cancer screening purposes. The device consists of two subsystems, both performing a non-invasive examination: the microwave breast imaging subsystem, and the optical breast contour detection subsystem. The device has been recently installed at the Clinical Research Facility of Galway University Hospital (CRFG) in Ireland, for a first-in-human pilot clinical test. In this paper, the methodology and indicative results from the on-site validation of the device, using anthropomorphic breast phantoms, are presented. The developed methodology is meant to be applied after each installation of the device in hospital, in order for the device functioning to be carefully verified and validated, before any patient recruitment is authorized.

The paper is structured as follows. All of the materials and methods used for the on-site validation of the imaging system are presented in Section 2 of the paper. In Section 2.1, a description of the Wavelia imaging system is provided. In Section 2.2, the fundamental working principles of the microwave breast imaging technology are summarized. In Section 2.3, the anthropomorphic breast phantoms which have been used for the design and testing of the Wavelia imaging system are presented. In Section 2.4, the main steps of the microwave breast imaging algorithm are outlined. In Section 2.5, an introduction to the optical scanner which is integrated in the Wavelia system is included; indicative results as used for the on-site validation of the optical scanner, are also shown in this subsection. In Section 2.6, the on-site validation test procedure used for the site acceptance of the Wavelia microwave breast imaging system is detailed. In Section 3, indicative validation test imaging results are shown. The presented results are grouped into two parts: in Section 3.1, images formed at a single vertical position of the sensor network are presented, whereas in Section 3.2, images formed using data collected at multiple vertical scan positions of the sensor network are shown. In Section 4, a summary of quality assessment (QA) results from the on-site validation of the system is included, followed by a short discussion on the potential sources of mismodeling of the breast with the available phantoms, which may inevitably lead to adjustments of the system when working with clinical data.

## 2. Materials and Methods

### 2.1. The Wavelia Microwave Breast Imaging System Prototype

**Figure 1:**
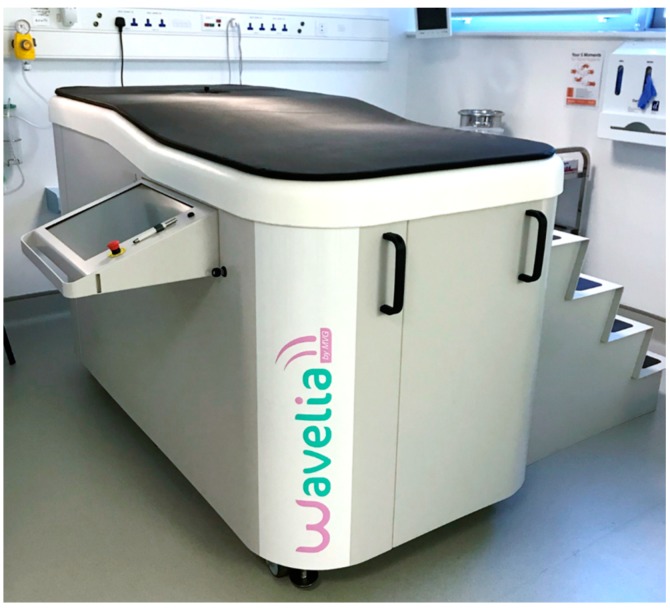
The Wavelia breast imaging system, which was recently installed in Galway University Hospital, for a first-in-human clinical test.

Wavelia is a prototype medical device that employs low-power electromagnetic waves for the detection of breast cancer. A photo of the device, as installed in the CRFG examination room, in Galway, Ireland for first-in-human pilot clinical testing, is shown in Figure 1.

As mentioned in the introduction, the device consists of the microwave breast imaging subsystem and the optical breast contour detection subsystem. The microwave breast imaging subsystem is an active device that illuminates the breast with non-ionizing low-power electromagnetic waves in the microwave frequency spectrum, which penetrate the breast under examination. The subsystem collects the scattered electromagnetic waves and recovers pertinent information about the breast tissue consistency based on the dielectric contrast of these tissues. The optical breast contour detection subsystem serves to provide the total volume and boundary contour of the breast, as a priori information for the microwave breast imaging subsystem.

During the examination, the patient will be lying in a prone position on the examination table. A dedicated circular opening on the examination table will permit the immersion of the breast in a specific liquid, which will serve as a coupling (transition) medium between the imaging system and the breast. The coupling liquid has been appropriately manufactured such that it has electromagnetic properties favoring the penetration of the electromagnetic wave in the breast.

The intended performance of the device is to unambiguously detect the presence of breast malignant lesions and estimate their 3D location within a given level of accuracy. While the ultimate goal is the diagnosis of breast cancer at an early stage of development, in the course of the pilot first-in-human trial, the achievable performance of the device will only be verified against benchmark cases of prediagnosed palpable cancers. To this extent, co-registration of the imaging results with available images from reference modalities (X-ray mammogram and/or ultrasound scans) will be performed. Thus, a “ground truth” will be available to assess the performance of the prototype device under test.

**Figure 2:**
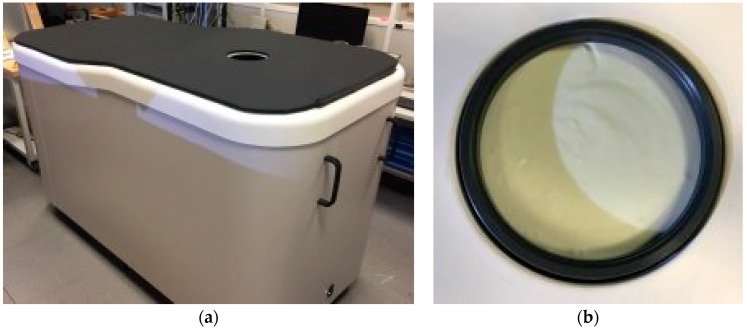
Wavelia microwave breast imaging system: (**a**) Top view of the examination table; (**b**) Zoomed view on the transition liquid in which the breast is immersed during the scan.

In Figure 2, a top view of the Wavelia microwave breast imaging subsystem examination table, as well as a zoomed view on the transition liquid in which the breast is immersed during the scan, are shown.

### 2.2. Microwave Breast Imaging at Prone Position: The Principle

The microwave imaging scan is performed using a network of 18 wideband Vivaldi-type antennas in a horizontal circular configuration. The sensors are located outside a container that hosts the coupling liquid. The sensors are piloted to perform a vertical motion such that the full breast volume is appropriately illuminated during the scan. The scan takes approximately 10 min for a breast of medium size, as the breast phantom used for the on-site validation of the system.

**Figure 3:**
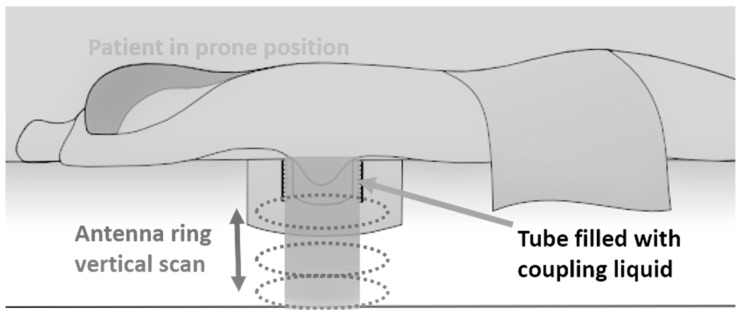
Microwave breast imaging examination: the principle.

A schematic description of the prone examination setup is shown in Figure 3.

The technology is very safe. The emitted microwave power level inside the breast is limited physically by the capacity of the radiofrequency components, such that the maximum radiated level inside the breast is always lower than 50 mW. Calculations have been performed for the localized specific absorption rate (SAR) in the breast. The maximum localized SAR in the breast complies with the International Commission on Non-Ionizing Radiation Protection (ICNIRP) recommendations and the European Union (EU) Directive 1999/519/CE on the limitation of exposure of the public to electromagnetic fields (compliance with a safety factor of four). The Radio-Frequency (RF) front end is based on vector network analyzer architecture. The resulting emission/reception RF chain has a dynamic range of 75 dB.

From the perspective of microwave imaging, the anatomy of the breast can be simplified to the following [14]:

- An adipose layer directly below the skin. This layer consists of vesicular cells filled with fat, which are aggregated into lobules and separated by Cooper’s ligaments;
- The mammary glands: the innermost tissue of the breast consists of about 15–20 sections, termed lobes, with many smaller sections of mammary glands, which are arranged in a circular fashion. These lobes and ducts are also surrounded by Cooper’s ligaments, which have the function of maintaining the inner structure of the breast and supporting the tissue attached to the chest wall;
- Posterior to the breast is the major pectoral muscle, as well as ribs two to six.

Breast tumors typically originate in the glandular tissue. The increased volume of water within the cancerous tissue is responsible for the strong electromagnetic scattering associated with microwave imaging. The increase of sodium and water, particularly inbound water within the tumor cells, leads to the greater conductivity and permittivity of the tumorous tissues [18,19].

Several studies have examined the dielectric properties of normal and cancerous breast tissue. Indicatively, in 1992, Campbell and Land measured in vitro the complex permittivity of female breast tissue at 3.2 GHz [20]. They reported a significant dielectric contrast between normal (fatty tissue and all other healthy breast tissues) and tumor tissue. They also suggested that due to the similarity in the dielectric properties of malignant and benign tumors, it might not be possible to distinguish between the two based on dielectric properties alone.

Some additional characteristics that are inherent to benign and malignant tumors have the potential to be helpful for tumor classification using microwave imaging, such as the tumor shape and surface texture [21,22,23]. Malignant tumors usually present the following characteristics: irregular and asymmetric shapes, blurred boundaries (lack of sharpness), rough and complex surfaces with spicules or microlobules, non-uniform permittivity, distortion in the structure of the breast, and irregular tissue density (due to masses and calcifications). Conversely, benign tumors tend to have the following characteristics: spherical, oval, or at least present well-circumscribed contours, compactness, and a smooth surface.

In the Wavelia microwave breast imaging device, multistatic radar detection technology [24,25] is employed. In multistatic radar imaging systems, each element of a fixed-element array illuminates the imaging scene in turn, while the other antennas record scattering at various angles from the transmitter boresight. Due to the spatial diversity of the receiving antennas, the multistatic approach acquires enhanced information about the scatterers, using received signals that propagate outwards via different routes. The number of illuminating paths is limited by the array geometry.

Due to the dielectric contrast between the different breast tissues at the microwave frequency range [19,20,26], back-scattered radar signals are physically generated. The received radar echoes are appropriately processed in order to detect and localize any significant scatterers (tumors) in the breast. An increased level of coherence of reflections originating from a given location results in the high intensity of the radar image at the given location in the breast, thus suggesting the presence of a significant scatterer.

Prior to radar imaging of the interior of the breast, pre-processing of the backscattered signals is performed to remove artifacts in order to accentuate the useful radar echoes of weak power level. The strong artifacts mainly consist of direct coupling between the antennas, skin reflections, and antenna reverberation. Following artifact removal, an effective radar-imaging algorithm is employed to unambiguously detect the presence of tumors and accurately localize them, while simultaneously suppressing clutter due to the normal heterogeneity of breast tissue.

Apart from using reflected microwave energy to reconstruct images of the breast, the radar target signatures may contain additional information on the shape, size, and other features of the tumor. This information could potentially be exploited for discrimination between benign and malignant lesions.

### 2.3. The Breast Phantoms

During the design phase, but also for the on-site validation, the imaging device has been deployed with phantoms, which simulate the real breast. These phantoms have been manufactured considering:

▪ Realistic breast shapes extracted from a publicly available database of real MRI breast images [27];
▪ The state-of-art knowledge in terms of dielectric properties of the breast normal and malignant tissues in the frequency range of interest [26,28,29,30];
▪ Realistic asymmetric tumor shapes and sizes [22,23,31];

The manufactured breast phantoms have been presented in further detail in [32,33], by A. Fasoula et al. 

The breast phantom repository, as published by the University of Wisconsin [27], has been used to define MRI-based realistic breast geometries. Based on this, rigid plastic molds have been 3D-printed for the breast outer surface, as well as for the segmented fibroglandular tissue in the breast MRI image, after the minimum required simplification, such that the geometry is printable in a limited number of compartments. For the imaging tests, both molds are filled with liquids mimicking the adipose and fibroglandular tissue [34]. Either liquid is poured in the corresponding mold compartment; the compartment walls are sufficiently thin to avoid significant impact on electromagnetic wave propagation.

Solid mixtures of graphite, carbon black, and urethane are used to manufacture the skin and tumor phantoms. The formula published in [35] by J.Garrett et al. has been slightly adjusted to achieve solid mixtures with appropriate dielectric properties mimicking the corresponding types of tissues.

**Figure 4:**
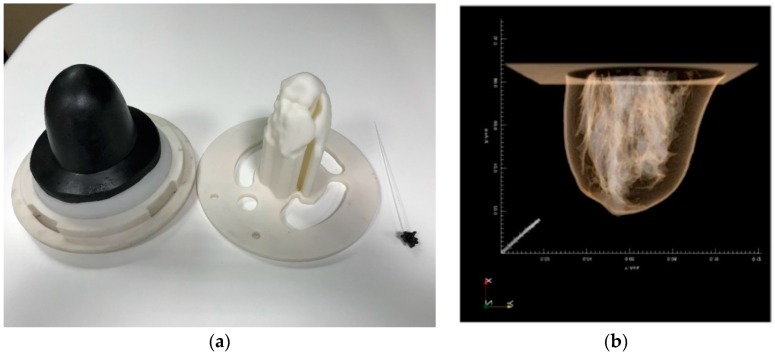
The breast molds: (**a**) Bottom view of the outer breast surface mold (on the left), covered with the black 2-mm thick skin phantom, and the outer fibroglandular tissue mold; (**b**) Original geometry of the breast, segmented from an MRI breast image.

In Figure 4, the geometry of one of the breasts of class ACR3 (heterogeneously dense) that has been selected from the database for the on-site validation of the imaging system, as well as the corresponding 3D printed molds, are depicted. The selected adipose tissue-mimicking liquid has a mean dielectric constant ε_r_ = 5, while the fibroglandular tissue-mimicking liquid has a mean dielectric constant ε_r_ = 36.

As depicted in Figure 4, a 2-mm thick skin layer with mean dielectric constant ε_r_ = 38 is attached to the breast outer surface mold. The selected material, apart from its adequate mean dielectric properties, also has a dispersive profile of complex permittivity well-fitting to the skin dielectric properties, as reported in the relevant literature [28].

A tumor is simulated by use of a microlobulated solid having a diameter of 14 mm and a mean dielectric constant ε_r_ = 52 within the frequency band of interest. The shape of the tumor phantom is based on a Gaussian random sphere (GRS) model of the breast lesions [21,22,23]. Aside from its adequate mean dielectric properties, the selected material used for this phantom has a dispersive profile of complex permittivity that fits well with the one of malignant breast tissue, as reported in the literature [29].

**Figure 5:**
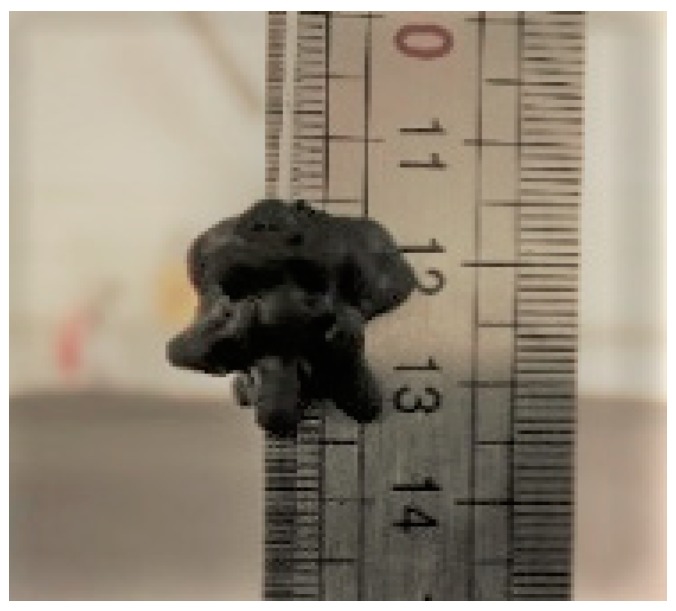
The tumor phantom: microlobulated shape, average radius of 14 mm, dielectric properties matching the measured dielectric properties of excised malignant tissue.

A photo of the tumor phantom used during the validation tests of the microwave breast imaging device is shown in Figure 5.

**Figure 6:**
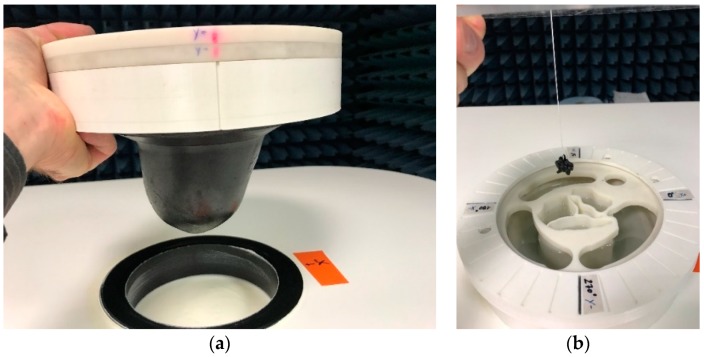
Preparation of the breast phantom for the microwave breast imaging test: (**a**) Immersion of the breast in the circular opening of the examination table, filled with transition liquid; (**b**) Inclusion of the tumor phantom in the fibroglandular tissue-mimicking liquid.

Two snapshots from the preparation of the experimental setup, before a typical validation test of the device, are shown in Figure 6.

### 2.4. The Imaging Algorithm

#### 2.4.1. The Physical Considerations and Modeling

**Figure 7:**
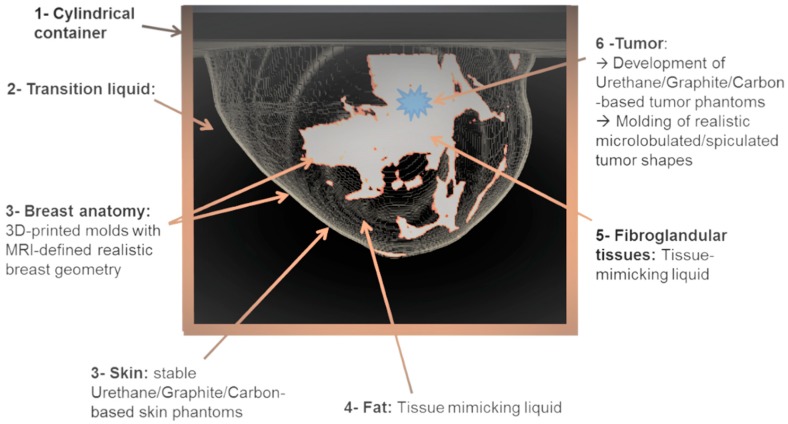
Realistic modeling of the near-field, non-planar, multi-layer, high permittivity transmission medium for electromagnetic wave penetration.

In order to properly design the data processing algorithms for such a device, it is fundamental to take into consideration the anatomy of the human female breast and translate it into an electromagnetic wave propagation problem to be resolved. As depicted in Figure 7, the breast skin layer, the fat, and the network of glandular tissue lobules and ducts of the human female breast have been considered to model the wave propagation path through the breast, before potentially reaching a tumor. As stated earlier, a network of sensors encircles a cylinder about which a vertical microwave imaging scan is performed.

The cylinder is filled with coupling transition liquid into which the breast is immersed. The transition liquid allows for optimizing the transmission of the electromagnetic waves from the antennas into the breast (similar function as for the gel used in ultrasound echography for optimizing the transmission of the ultrasound waves from the probe to the interior of the body). Thus, the transition liquid has been designed to have real permittivity that well matches the permittivity of the human skin, as specified by Lazebnik et al. [28]. At the same time, the conductivity of the liquid has been designed to be such that it introduces non-negligible propagation losses, thus mitigating the strong multipath waves that propagate in the cylinder without ever entering the breast, as initially suggested in [36] by P. Meaney et al. The real permittivity of the transition liquid ranges between 25 and 30, and its conductivity ranges between 0.2 S/m and 1.2 S/m in the working frequency band F = [1–4] GHz. The liquid is based on organic oil and deionized water mixed at a given proportion such that the desired dielectric properties are achieved.

Given the above considerations, the data processing algorithms of such a device should be designed such that useful information for breast imaging is acquired if the electromagnetic wave that is emitted from a sensor is received by another sensor of the network in a bistatic configuration. This step comes after transition from the following chain of non-planar layers with distinct dielectric properties (real permittivity and conductivity), which are each:

Transmitting antenna → Cylinder → Transition liquid → Skin → Fat → Glandular tissue → Tumor
Receiving antenna ← Cylinder ← Transition liquid ← Skin ← Fat ← Glandular tissue ← Tumor

The contrast in terms of the dielectric properties of the consecutive layers is responsible for the intensity of the echoes that are generated due to the transition of the electromagnetic wave via the respective layers. Thus, a significant tumor echo would be evoked that is conditioned on sufficient dielectric contrast between the normal glandular tissue and the cancerous tissue.

In addition, both the breast tissue and the transition liquid are materials of non-negligible conductivity that introduce noticeable radar wave propagation losses. This means that even if sufficient dielectric contrast exists to evoke significant reflection from tumors, the propagation losses along the path between the sensors and the tumor will lead to reflected signals of weak intensity compared to the unwanted reflections originating closer to the sensors. Namely, it is the interaction (coupling) between the antennas themselves, as well as the reflections that are generated by the skin layer once the electromagnetic wave impinges on the external surface of the breast, which represent signals that are several orders of magnitude larger in intensity than the weak reflections originating from the interior breast tissues.

Given the above principles, which are related to the physical nature of the problem, an imaging algorithm that is carefully customized for the application has been designed.

#### 2.4.2. The Data Pre-Processing Steps

Several pre-processing steps are applied to the data measured by each couple of transmitting/receiving antennas, before this data can be efficiently used for imaging. The objective of the pre-processing steps is to mitigate the strong coupling between the antennas and the strong interference originating from the skin and other interwall reflections close to the breast surface. In the actual experimental setup, the effective employment of the data pre-processing steps reveals useful radar target echoes 30–40 dB below the raw measured data power level. However, the data pre-processing steps, being directly linked to the nature of the measured signal, are susceptible to evolving once the imaging system is employed in the clinical setting.

- Data calibration at the presence of the breast

As a first step, drift correction, with respect to a reference channel, is applied to the raw data measured by each couple of transmitting/receiving antennas; any time-varying drifts are thus eliminated before further processing of the signal.

The presence of the breast at a close vicinity to the sensor network significantly modifies the measured coupling between sensors. For this reason, a calibration process is employed to dynamically estimate the coupling signal based on a bunch of data from the scan that has been measured at similar conditions. The data-driven estimation is performed in the frequency domain at each vertical scan position and for each T*x*/R*x* couple in the network. The estimated coupling signal $DCal_{Txi/Rxj,H_{n}}\left(f\right)$, is further subtracted from the drift-corrected raw data $Dat_{DriftCorr,Txi/Rxj,H_{n}}\left(f\right)$.

A multiplicative compensation factor $PhCen_{Corr,Txi/Rxj}\left(f,e_{r,trans}\left(f\right)\right)$ is then applied to the calibrated data in order to geometrically align the data. The phase-center compensation term is computed for each T*x*/R*x* couple in the network for each operating frequency point and is subject to the dielectric constant $e_{r,trans}\left(f\right)$ of the transition liquid, as a function of the frequency. Conditioned on temperature preservation in the operating limits of the device, such that the transition liquid dielectric properties are known, this term does not require dynamic data-driven estimation; it is a priori defined and stored during the system characterization at factory.

(1) $$Dat_{CAL,Txi/Rxj,H_{n}}\left(f\right)=\left(Dat_{DriftCorr,Txi/Rxj,H_{n}}\left(f\right)-DCal_{Txi/Rxj,H_{n}}\left(f\right)\right)\cdot PhCen_{Corr,Txi/Rxj}\left(f,e_{r,trans}\left(f\right)\right)$$

- Reconstruction of the breast external envelope

This estimation module uses as input a reduced set of data from the microwave breast imaging system, which after calibration for removal of the strong antenna coupling, is used to reconstruct the external surface of the breast with limited accuracy. The calibrated data is used in conjunction with an active contour model to estimate a simple closed contour representing the skin return boundary, based on bistatic wave-front detection, at each vertical scan position. The algorithm has been presented in more detail in [37] by P. Lawrence et al.

- Independent Component Analysis, in the frequency domain

The independent component analysis (ICA) is a well-known method for finding underlying factors, or components, from multivariate statistical data [38,39]. The ICA method has been used extensively in various application domains, among which medical imaging is included, for feature extraction and selection, or even pathology identification [40,41].

What distinguishes ICA from other methods is that it looks for components that are both statistically independent and non-Gaussian. Given a set of observations of stochastic processes $x_{1}(t),x_{2}(t),\ldots ,x_{m}(t)$, where t denotes the sample index, assume that they are generated as a linear mixture of independent components y = W·x, where W is some unknown matrix. Independent component analysis consists of estimating the mixing matrix W, such that the non-Gaussianity of the components y_i_(t) is maximized. The kurtosis and the negentropy are two of the most commonly employed measures of non-Gaussianity for estimating the mixing matrix W [38].

In the case of radar signals, ICA can be performed either in the time domain, or in the frequency domain [42,43,44,45]. In our data processing chain, we have opted for the frequency-domain ICA, applied to the calibrated data $DCal_{Txi/Rxj,H_{n}}\left(f\right)$ per T*x*/R*x* couple at each vertical scan position H_n_.

Segmentation of the data vector in frames of appropriate length, via application of a sliding window in frequency, is initially applied. Principal component analysis (PCA) is subsequently performed for data pre-whitening and dimensionality reduction [46], prior to input into the ICA algorithm. The selected sliding step in frequency is an important parameter that is directly linked to the spectral properties of the underlying signal and the principal modes to be preserved after pre-processing. The ICA operation is denoted in Equation (2), where $Dat_{PCA-TAB,Txi/Rxj,H_{n}},\;\left(M\cdot N_{f}\right)$ is the block of M principal modes that is provided as input to the ICA algorithm, $Dat_{ICA-TAB,Txi/Rxj,H_{n}},\;\left(M\cdot N_{f}\right)$ is the block of M ICs, as estimated by the algorithm, M corresponds to the number of sliding windows in the frequency that is initially selected, and N_f_ is the number of frequency samples in the measured data vector.

(2) $$Dat_{ICA-TAB,Txi/Rxj,H_{n}}=W^{H}\cdot Dat_{PCA-TAB,Txi/Rxj,H_{n}},\forall H_{n}\;\mathrm{and}\;{\mathrm{Tx}}_{i}/{\mathrm{Rx}}_{i}$$

- Data filtering: IC Selection with Appropriate Spectral and Geometry-based Features

The clear function of the ICA data pre-processing step is to classify/separate useful against interference (strong clutter components), based on:

▪ The distinct spectral properties of the various radar target echoes i.e., frequency dispersion is normally translated to higher kurtosis [47,48].
▪ The estimated location from which each IC radar echo originates: an inverse fast Fourier transform (IFFT) for transformation of the IC from the frequency domain to the time domain is applied for this purpose; the correspondence between time and distance is established using as input the prior estimate of the breast contour, the known dielectric properties of the transition liquid, and an assumption on the average dielectric properties in the interior of the breast (directly derived from an assumption on the percentage of fibro-glandular versus adipose tissue in the breast).

Given the above considerations, two filtering steps are sequentially applied to the data:

▪ Filtering-out ICs with spectral profile incompatible with radar target echoes originating from the breast tissues, given the expected level of frequency dispersion; in the future, additional pattern features may be identified and employed at this filtering step, based on measurements with real breast tissues.
▪ Filtering-out ICs that are associated with radar target echoes originating from either very short distances (residual coupling) or very long distances (multipath) with respect to the sensors; the ICs that are filtered out at this step cannot physically correspond to the breast tissues, in terms of geometry.

- Propagation Loss Compensation

In order for the imaging algorithm to work properly, it is important to compensate for the electromagnetic wave propagation losses, which vary significantly along the working frequency band in the case of the highly-dispersive breast tissues.

Given the estimate of the distance from which the radar target echo that is associated with each IC originates (as estimated for the purpose of the distance-based filtering), a multiplicative propagation loss compensation term that is both frequency and distance dependent is applied to each IC. A characterization of the propagation loss model, which is applicable to the specific near-field radar imaging setup, is required to perform a good compensation. For now, an estimate, which is planned to be further refined in the future, is applied which achieves partial compensation of the propagation losses.

The energy focusing level, which is retrieved on the images, is expected to be degraded in the case of target sources for which the propagation loss compensation has not been properly performed at this pre-processing step. The propagation loss compensation term being dependent on the distance between the sensors and the target location to which each IC is associated means that it is also dependent on an assumption of the percentage of fibroglandular tissue $pc_{fib}$ present along the specific bistatic radar path.

Given all of the above considerations, a filtered version of $DCal_{Txi/Rxj,H_{n}}\left(f\right)$ is reconstructed using the ICs obtained from the two filtering stages. A multiplicative propagation loss compensation term is applied separately to each IC before concatenation.

(3) $$\mathrm{DCal}-{\mathrm{Filt}}_{\mathrm{Txi}/\mathrm{Rxj},H_{n}}(f)=\sum_{i\in \{IC_{\mathrm{rem}}\}}{\mathrm{Dat}}_{\mathrm{ICA}-\mathrm{TAB},\mathrm{Txi}/\mathrm{Rxj},H_{n}}(i,f).\;\mathrm{LossComp}(f,d_{i},pc_{\mathrm{fib}}),\;\forall H_{n}\;\mathrm{and}\;{\mathrm{Tx}}_{i}/{\mathrm{Rx}}_{i}$$

In Equation (3), IC_rem_ denotes the set of IC indices that have been maintained after the two-step filtering, while d_i_ denotes the bistatic radar distance of the target echo that has been associated with the i^th^ IC.

#### 2.4.3. The TR-MUSIC (Time-Reversal Multiple Signal Classification) Imaging Algorithm

After pre-processing the signals, as measured by various combinations of transmitting/receiving antennas—thus in various bistatic configurations—are combined in a multistatic radar imaging algorithm to generate an image of the interior breast tissues. The combination of multistatic radar paths in the same imaging algorithm enhances the angular diversity of the input information, thus making the algorithm more robust against clutter (unwanted distributed interference echoes from the interior of the breast) and enhancing the focusing of the image energy on small pronounced targets.

The imaging algorithm that is used is the time-reversal multiple signal classification (TR-MUSIC) algorithm, which was originally conceived for the detection of obscured radar targets in heavily cluttered environments, in the case of surveillance and tracking defense radars [49]. The original definition of the algorithm works optimally for a finite collection of point targets, as is the case when small targets are observed by a radar with limited spatial resolution, or when the first-order Born approximation is valid for the scattering mechanisms that dominate the imaging scene [50]. Further studies have been subsequently performed to generalize the algorithm in cases of multiple scattering phenomena [51] or extended targets, as is the case when a target is large relative to the size of the radar resolution cell [52]. More recently, the algorithm has been also proposed for breast cancer detection in dense breasts [53,54,55,56,57,58], albeit limited to simulations and no experimental data.

The main steps of our implementation of the algorithm are outlined as follows:

▪ A limited number of $N_{\mathrm{fsel}}$ frequency points is selected from the total of measured frequency points in the operating band.
▪ Sectorization is performed, such that multiple images are formed at each frequency and each vertical position of the sensor network, each time using a different sector of the circular network. The selected number of sensors in the sector is further denoted as $N_{s}$. The total number of sectors required to scan over the full 360° around the breast is denoted as $N_{\mathrm{sect}}$.

Both the selection of specific frequency points, and the physical size and number of elements in the sub-arrays (sectors) used for the elementary image formation, can be critical to the achievable system performance in terms of unambiguous target (tumor) detection in the breast.

Monochromatic (single frequency) images are formed for each selected frequency point and each sector of sensors as follows:

- The multistatic frequency response matrix (MFRM) is formed using the calibrated and filtered data at the specific frequency:
  (4) $$S_{sect}\left(f\right)=\left[\begin{array}{cccc} S_{Tx_{1}/Rx_{1}} & S_{Tx_{1}/Rx_{2}} & \ldots & S_{Tx_{1}/Rx_{N_{s}}}\\ S_{Tx_{2}/Rx_{1}} & \ddots & \ddots & \vdots \\ \vdots & \ddots & \ddots & \vdots \\ S_{Tx_{N_{s}}/Rx_{1}} & \ldots & \ldots & S_{Tx_{N_{s}}/Rx_{N_{s}}} \end{array}\right],\;f=1:N_{fsel}$$
  where:
  (5) $$S_{Tx_{i}/Rx_{j}}(f)=DCal-Filt_{Txi/Rxj,H_{n}}(f),\;\forall H_{n}\;\mathrm{and}\;{\mathrm{Tx}}_{i}/{\mathrm{Rx}}_{i}$$
  as defined in Equation (3).
- The time-reversal operator is subsequently formed as:
  (6) $$T_{\mathrm{sect}}(f)=S_{\mathrm{sect}}{\left(f\right)}^{H}\cdot S_{\mathrm{sect}}(f)$$
  with H denoting the Hermitian transpose.
- Eigenvalue decomposition is performed on $T_{\mathrm{sect}}(f)$, and an appropriate model order selection criterion is used to separate the resulting eigenspace into signal and noise subspaces [59]:
  (7) $$\left\{\mathrm{Signal}\;\mathrm{subspace}:\;\{\lambda_{s},Q_{s}\}={\left\{\lambda_{i},Q_{i}\right\}}_{i=1}^{M_{ord}},\;\mathrm{Noise}\;\mathrm{subspace}:\;\{\lambda_{N},Q_{N}\}={\left\{\lambda_{i},Q_{i}\right\}}_{i=M_{ord}+1}^{N_{s}}\right\}$$
  where M_ord_ is the selected model order. The separation can be a challenging task, if in the imaging scene there are multiple interacting non-point targets, as is typically the case for breast imaging. The effective separability between the signal and noise subspace has a significant direct impact on the final imaging result, given that the principle for the formation of this type of image is the orthogonality between the two subspaces.
  (8) $$Q_{S}^{H}\cdot Q_{N}=0$$
- The image, or the so-called TR-MUSIC pseudospectrum at the pixel p and the frequency f, when using the sector of sensors sect at the vertical scan position h_j_, is formed as:
  (9) Imsect,hj(p,f)=1‖(QNH⋅Gsect(p,f))H⋅(QNH⋅Gsect(p,f))‖
  where:
  (10) $$G_{sect}\left(p,f\right)={\left[\begin{array}{cccc} g_{0}\left(p_{TRx_{\mathrm{sect},1}},p,f\right) & g_{0}\left(p_{TRx_{\mathrm{sect},2}},p,f\right) & \ldots & g_{0}\left(p_{TRx_{\mathrm{sect},N_{s}}},p,f\right) \end{array}\right]}^{T}$$
  is the illumination vector of the sector sensor array sect at the frequency f and the pixel location p in the imaging zone.

In Equation (10), $g_{0}\left(p_{TRx_{\mathrm{sect},i}},p,f\right)$ denotes the elementary Green function (i.e., the impulse response function of the propagation path) from the individual antenna at position $p_{TRx_{\mathrm{sect},i}}$ to the arbitrary point p in the scanning region at the frequency f, while T denotes the matrix transpose.

The TR-MUSIC pseudospectrum in Equation (9) gets maximized, thus highlighting a target presence, at the pixel location p, at which the orthogonality constraint between the sensor array illumination vector and the signal noise subspace is better met.

This arises from the assumption that a linear decomposition of the illumination vector $G_{sect}\left(p,f\right)$ in the signal subspace Q_s_ exists such that:

(11) $$G_{sect}\left(p,f\right)=Q_{s}\cdot B^{\left(m\right)},\;\mathrm{with}\;B^{\left(m\right)}={\left[\begin{array}{cc} b_{1}^{m} & b_{2}^{m}\cdots b_{M_{ord}}^{m} \end{array}\right]}^{T}\;a\;\mathrm{set}\;\mathrm{of}\;\mathrm{linear}\;\mathrm{coefficients}$$

and the orthogonality constraint in Equation (8).

#### 2.4.4. The Composite Image Formation

The monochromatic (single-frequency) image, as defined in Equation (9), may be difficult to be exploited as such for unambiguous and comprehensive interpretation of the imaging scene, due to inevitable corruption of the signal by residual noise and interference, even after pre-processing. Frequency diversity is commonly employed to mitigate the presence of frequency-dependent clutter (unwanted interference) radar echoes. The multi-frequency TR-MUSIC image at the sector sect and the vertical scan position h_j_ is defined in Equation (12):

(12) Imsect,hj(p)=∑f=1Nfsel1‖(QNH⋅Gsect(p,f))H⋅(QNH⋅Gsect(p,f))‖

In order to assure visibility of the breast over the full azimuth domain of 360°, integration is performed on multiple partial images, computed per sectors of sensors all around the breast. The composite image that is formed using all the $N_{\mathrm{sect}}$ elementary multi-frequency images at a given vertical position of the sensor network is defined in Equation (13):

(13) ImTOT,hj(p)=1Nsect⋅∑i=1NsectImsect,hj(p)

The composite image of Equation (13) is the first type of image that is used for the validation of the imaging system using a well-controlled breast phantom, imaged at a single vertical position of the sensor network, in the vicinity of the tumor phantom.

Integration of multiple partial images of the complete imaging scene, computed all along the vertical scan of the sensor network, is further applied to form the full 3D image of the breast.

The composite image using data from multiple vertical scan positions of the sensor network is defined in Equation (14):

(14) ImTOT,MultiH(p)=1Nh⋅∑j=1NhImTOT,hj(p)

where $N_{h}$ is the number of vertical scan positions of the sensor network that are used to form the full 3D image.

#### 2.4.5. The Focusing Metrics, as a Means of Adjustment of the Breast Mean Permittivity

In order to map the multistatic radar echoes to the imaging grid under investigation, a model for the electromagnetic wave propagation modes is employed, as defined in Equations (9) and (10).

In the actual version of the microwave imaging device, propagation in two homogeneous lossless media is considered in the model. Lossless media are justified, given that loss compensation has been applied to the pre-processed signals before entering the imaging algorithm, as defined in Section 2.4.2.

Separation of the space in two media is assumed, given that the heterogeneous distribution of the tissues in the interior of the breast is unknown and sought to be estimated by the imaging algorithm. Thus, the two media that are provided as a priori to the imaging algorithm are: the transition liquid between the antennas and the exterior breast surface, and then the interior of the breast associated with an “average” dielectric permittivity, which remains homogeneous per coronal slice of the breast. The breast external surface has been estimated prior, using a subset of the calibrated data, as mentioned briefly in Section 2.4.2; this information is exploited here to define the border between the two distinct media of propagation.

The elementary Green function $g_{0}\left(p_{TRx_{\mathrm{sect},i}},p,f\right)$ involved in Equation (10) is further defined as:

(15) $$g_{0}\left(p_{TRx_{\sec t,i}},p,f\right)=j\cdot H_{0}^{\left(1\right)}\left(k_{bg}\left(f\right)\cdot \left\|p-p_{TRx_{\mathrm{sect},i}}\right\|+D{\hat{k}}_{breast}\left(f\right)\cdot {\hat{d}}_{InBreast,i,p}\right)$$

where:

▪ $H_{0}^{\left(1\right)}$ is the Hankel function of first kind and zero order: $H_{m}^{\left(1\right)}\left(x\right)={\left(-j\right)}^{m+1}\cdot \frac{e^{j\cdot x}}{x},\;\mathrm{with}\;m=0$,
▪ $k_{bg}\left(f\right)=\frac{2\pi f}{c_{0}}\cdot \sqrt{e_{r,trans}\left(f\right)}$ is the wavenumber for propagation in the transition liquid,
▪ $D{\hat{k}}_{breast}\left(f\right)=\frac{2\pi f}{c_{0}}\cdot \left(\sqrt{{\hat{e}}_{r,InBreast}\left(f\right)}-\sqrt{e_{r,trans}\left(f\right)}\right)$ is an ‘average’ differential wavenumber for propagation in the breast,
▪ $c_{0}$ is the speed of light in vacuum,
▪ $e_{r,trans}\left(f\right)$ is the known dielectric constant of the transition liquid,
▪ ${\hat{e}}_{r,InBreast}\left(f\right)$ is an estimate of the average equivalent dielectric constant of the breast, and
▪ ${\hat{d}}_{InBreast,i,p}$ is an estimate of the propagation path in the breast, in the case of a wave propagating from the sensor $TRx_{\mathrm{sect},i}$ to the pixel p, knowing the wavefront corresponding to the external surface of the breast.

The “average” equivalent dielectric constant of the breast is defined in Equation (16) as a function of the dielectric constant of the adipose and fibroglandular tissue, mixed at proportion $pc_{fib}$.

(16) $${\hat{e}}_{r,InBreast}\left(f\right)=\left(pc_{fib}\cdot {\hat{e}}_{r,fibroglandular}\left(f\right)+\left(1-pc_{fib}\right)\cdot {\hat{e}}_{r,adipose}\left(f\right)\right)\cdot 10^{-2}$$

**Figure 8:**
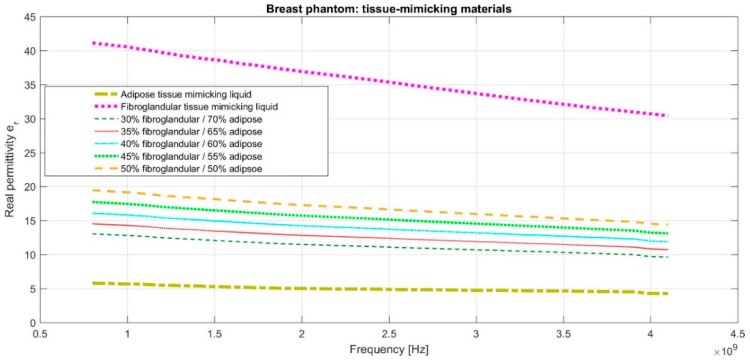
“Average” equivalent dielectric constant along a bistatic path through the breast, under various assumptions on the percentage of fibroglandular tissue $pc_{fib}$. Applicability to the breast phantoms used for the design validation of the microwave breast imaging system.

${\hat{e}}_{r,InBreast}\left(f\right)$ is plotted in Figure 8 for various assumptions $pc_{fib}$, while considering for the adipose and fibroglandular tissue dielectric properties the ones of the corresponding tissue-mimicking liquids used to fill the breast phantom molds of the Wavelia microwave breast imaging system, as defined in Section 2.3.

Parametric images are generated under varying assumptions of percentage of fibroglandular tissue $pc_{fib}$ along the propagation path from a given transmitting antenna, to the breast and back to a given receiving antenna. The parameter $pc_{fib}$ impacts on both the estimate of the lossless elementary Green function $g_{0}\left(p_{TRx_{\mathrm{sect},i}},p,f\right)$, as defined in Equation (15), but also on the computation of the propagation loss compensation term LossComp $(f,d_{i},pc_{fib}),$ in Equation (3) of the data pre-processing chain.

The generated set of parametric images is further evaluated in terms of focusing, using appropriate image focusing measures [60,61,62]. The optimal $pc_{fib}$ assumption is automatically selected based on maximization of the focusing capability of the imaging algorithm, under the specific $pc_{fib}$ assumption. Given the varying consistency of the heterogeneous breast along the vertical scan, the focusing operation is performed per vertical position of the sensor network, thus on the image type defined in Equation (13).

For the analysis presented in this paper and used for the on-site validation of the Wavelia imaging system before its pilot clinical test, the image curvature, as defined in [60] by S. Pertuz et al., is used as the focusing metric (FM) for the parametric images. The intensity of the TR-MUSIC pseudospectrum of Equation (13) is interpolated by means of a quadratic surface $f(x,y)=c0\cdot x+c1\cdot y+c2\cdot x^{2}+c3\cdot y^{2}$, where the vector of coefficients C = [c0 c1 c2 c3]^T^ is computed through least squares by applying two convolution masks, as defined in [60] by S. Pertuz et al. The curvature of the quadratic surface is used as the focusing metric (FM) for the image:

(17) FM=|c0|+|c1|+|c2|+|c3|

The quadratic surface fitting and FM computation is actually performed per regions of interest (ROIs) of limited size on the image. The selected ROI size is related to the image resolution, as well as the size of detectable scattering objects in the radar imaging scene. The maximal image curvature (FM) over all of the ROIs is computed per parametric image. The $pc_{fib}$ associated with the image with overall maximal curvature is selected as optimal at a given vertical section of the breast (coronal breast size) in front of the sensor network. The composite multi-height image, as defined in Equation (14), is automatically formed via concatenation of all the coronal slices with maximal curvature (FM).

At the current stage of system development, the image formation is performed offline. It may take a few hours for the focusing algorithm to run the multiparametric (multi $pc_{fib}$) multi-sector images for all the vertical (coronal) slices of the breast. The total duration for the composite image formation will depend on the size of each breast (= i.e., number of coronal slices to be processed) and the number of assumptions on the background breast permittivity under test (=size of the parameter set $pc_{fib}$).

The actual implementation is valid, as such, in the case of a single dominant target (tumor) in each coronal slice of the breast. Both the breast phantoms and the clinical setting for the pilot first-in-human testing of the device are compliant with such a physical assumption. Appropriate complexification of the algorithm is planned for the near future in order to properly handle the realistic case of multiple lesions being present, sought to be detected, and accurately localized per coronal slice of the breast.

**Figure 9:**
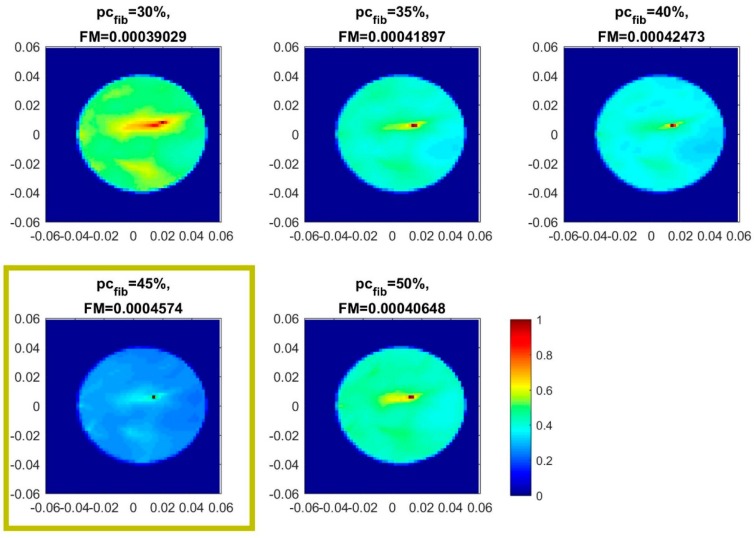
Example of focusing evaluation on a coronal breast slice. Parametric images generated for five assumptions in terms of percentage of fibroglandular tissue in the breast. Optimal $pc_{fib}$ = 45%, automatically selected based on maximization of the focusing metric (FM). Single tumor (dominant scatterer) detected on the specific breast coronal slice.

An example of the computed FM for a set of five $pc_{fib}$-parameterized images, as well as the result of optimal $pc_{fib}$ selection, is shown in Figure 9. The FM values are appropriately rescaled by the algorithm, such that the resulting values are comparable among various coronal cross-sections of the breast. The depicted images are normalized to maximum intensity.

### 2.5. The Optical Breast Scan and Metrology

As mentioned in Section 2.1, the Wavelia medical device consists of two subsystems, both performing a non-invasive examination: the microwave breast imaging subsystem, which is the main part of the system, and the optical breast contour detection subsystem, which plays an auxiliary role. The objective of the optical subsystem is triple:

▪ Compute the volume of the patient’s breast, thus indirectly deriving the required volume of transition liquid such that the container of the microwave breast imaging subsystem is optimally filled after immersion of the breast;
▪ Compute the vertical extent of the pendulous breast, in order to optimally dimension the vertical scan of the microwave breast imaging system;
▪ Reconstruct fully the external envelope of the breast, with high precision; such information will further serve to control the potential level of deformation of the breast due to immersion in the transition liquid during the microwave imaging scan. It may also serve as an intermediate step when registering the 3D microwave image with reference to the 2D mammographic projections of the patient’s breast, for comparison and validation of the microwave breast imaging modality.

The optical scan of the breast will be performed just before the microwave imaging scan, during the clinical testing of the Wavelia system. In order for the optically reconstructed breast envelope to be useful a priori information for the microwave imaging system, it is important that the patient is lying in the same prone position during both examinations. Thus, an identical examination table as the one used for the microwave imaging and shown in Figure 1, is integrated with the optical breast contour detection subsystem as well.

The patient is lying on the examination table, with her breast under examination inserted in the circular opening of the examination table. For this examination, there is no coupling liquid, as shown in Figure 2 for the microwave imaging system. The breast is in the air, hanging below the examination table. A 3D infrared camera is placed below the examination table at a distance of several tens of centimeters below the breast. A motorization system enables the azimuthal motion of the camera in one single horizontal plane. The azimuthal scan of the 3D camera permits reconstructing the external envelope of the breast with sub-millimetric precision.

**Figure 10:**
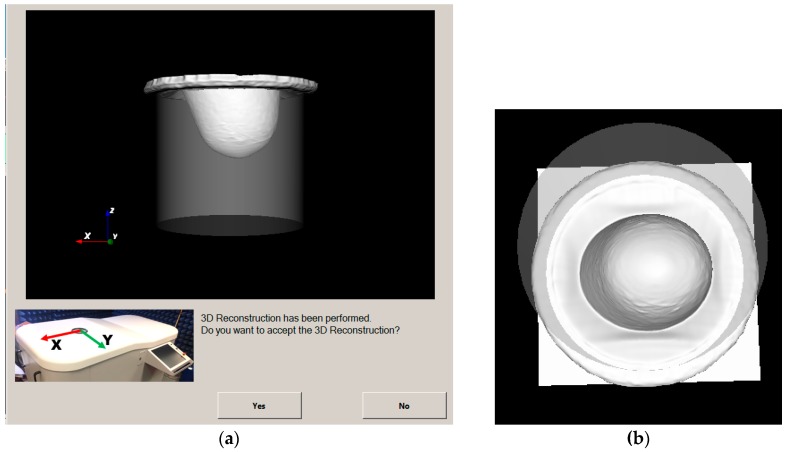
Reconstructed outer surface of the breast phantom #1 (**a**) Screen capture from the Wavelia Optical Breast Contour Detection subsystem, as provided to the user, (**b**) Zoomed bottom view of the reconstructed outer surface of the breast.

In Figure 10, the reconstructed outer surface for the breast phantom that has been specified in detail in Section 2.3 and is used for the validation of the Wavelia imaging system on site is shown. Both a side view and a bottom view are indicatively shown, as provided to the system user for acceptance of the scan.

**Figure 11:**
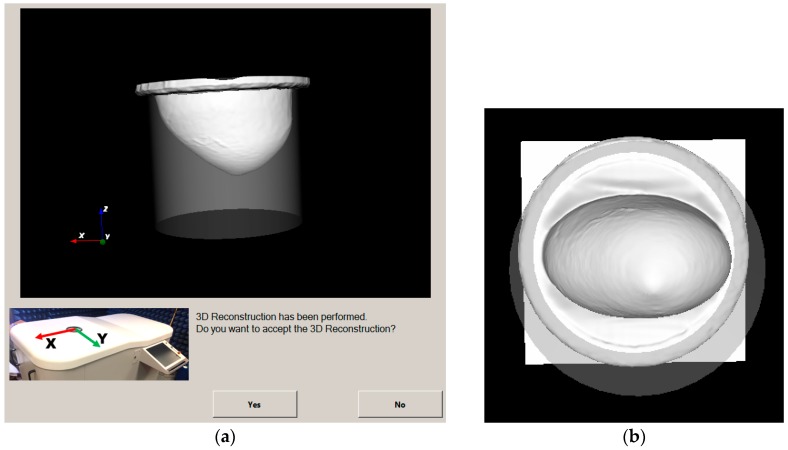
Reconstructed outer surface of a second breast phantom #2, of significantly bigger size, (**a**) Screen capture from the Wavelia Optical Breast Contour Detection subsystem, as provided to the user, (**b**) Zoomed bottom view of the reconstructed outer surface of the breast.

In Figure 11, the reconstructed outer surface of a second breast phantom of different shape and a significantly bigger size is illustrated.

In Table 1, the measurement results for both breast phantoms are given, for one optical scan performed at factory and another scan performed after the installation of the system on site. Reproducible results have been achieved with very good accuracy; these results served for the site acceptance of the optical system at the hospital.

The achievable level of accuracy for both the computation of the breast volume and the computation of the vertical extent of the pendulous breast is compatible with the expected values and independent of the breast size and shape, as long as the breast is within the limits of acceptable sizes specified in the clinical protocol NCT03475992 [63].

### 2.6. The On-Site Validation Test Procedure for the Microwave Breast Imaging System

#### 2.6.1. Controlled Environmental Conditions for Nominal Operation of the Imaging System

At this stage of prototype development, the imaging system is required to operate in a controlled environment, for the nominal system performance to be assured. The examination room temperature should range between 20–25 °C during the full examination, which takes approximately 1 h, including: the optical and microwave scan of both breasts of the patient, all the intermediate system preparation steps, the transition liquid preparation steps, and the system quality checks.

In order to assure compliance with these temperature limits during the examination, it is recommended that the room temperature does not exceed 21–22 °C at the beginning of the examination.

For the system on-site validation tests with breast phantoms, the temperature is monitored both at the beginning and at the end of each test. The monitoring is performed at the following control points:

▪ container filled with transition liquid: measurement at the center and close to the borders of the container
▪ breast mold compartments filled with fibroglandular tissue-mimicking liquid: measurement at three different points, or compartments

In Table 2, the temperature monitoring data for an on-site system validation test, which has been marked as compliant with the nominal operating conditions, is indicatively provided.

#### 2.6.2. System Stability Verification

A series of systematic tests are regularly performed at system installation in order to assess the repeatability of the measuring capability of the system. The assessment of the repeatability before performing a RF scan is fundamental to assure that a reliable and exploitable measurement can be performed.

A procedure for quantitative assessment of the system reliability has been developed. A reduced version of this is also performed automatically by the system before the examination of each patient. It consists of repeating a dummy (no breast immersion) measurement several times and performing three tests to quantify the level of variability of the complex measurements, both in terms of amplitude and phase.

- Verify that the amplitude envelope of the raw measured data keeps consistent with the lower and upper-level masks, as predefined at factory;
- Perform first and second-order statistics on raw measured data after drift correction: evaluate the stability, both in amplitude and phase, of the reference channel
- Perform first and second-order statistics on calibrated data: evaluate the multi-run stability, both in amplitude and phase, on a limited set of T*x*/R*x* couples.

#### 2.6.3. Imaging Test with Complex Breast Phantom at Two Azimuthal Rotational Positions

For the on-site validation of the system imaging performance after installation, a controlled test with a complex breast phantom is performed. A tumor phantom is included at a given known position in the breast. Quantitative evaluation of a series of metrics is performed for the quality assessment and validation of the scan. For this reason, it is important that repeated testing with the exact breast and tumor location configuration has been prior performed and thoroughly characterized at the factory. The breast and tumor phantoms that are used for the on-site system validation have been defined in Section 2.3.

The scan is repeated for two distinct azimuthal rotations of the phantom (azimuthal rotation of both the breast and tumor by 180°, such that the relative location of the tumorous inclusion in the breast remains constant). The purpose of the breast rotation is to identify and characterize any “non-symmetries” in the system imaging performance, due to residual uncalibrated imperfections of the system circular network. For the definite on-site validation of the system after installation, a follow-up of the system imaging performance, as evaluated on the two azimuthal rotations of the breast phantom, is performed over several days.

After the system acceptance on site, and while the pilot clinical test is running on patients, the scan of the two breast phantom positions is recommended to be repeated and evaluated at regular intervals in time (e.g., regular monthly, or bi-monthly, interventions by the device manufacturer on site for control and maintenance). It is important to put into place such a regular follow-up in order to better assure the pilot clinical trial data quality, using a system at the prototype level of development.

**Figure 12:**
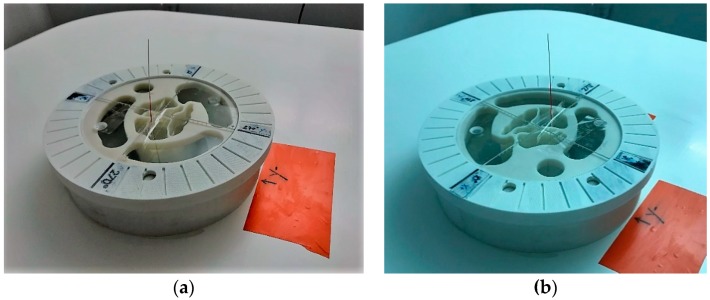
Breast phantom ready for the microwave imaging test: examination table top view (**a**) Breast phantom rotational position #1; (**b**) Breast phantom rotational position #2.

In Figure 12, a top view of the Wavelia examination table, after installation of the breast phantom for the regular validation test, is shown. The breast phantom is maintained at the known position, using a supporting ring structure. The tumor is inserted at the predefined 3D location, using a rigid string of known length, inserted via a hole at a precisely known (*x*, *y*) position on the phantom support structure. A photo of the two azimuthal rotation positions of the phantom, as used for system validation, is shown in Figure 12a,b.

#### 2.6.4. Centering Assessment of the Reconstructed Breast Outer Surface

The breast-centering quality test is performed each time on a single breast contour that is associated with a single vertical scan position of the sensor network predefined by the user. A coronal slice close to the middle vertical extent of the pendulous breast is normally selected for the evaluation of the centering of the breast with respect to the imaging zone.

Given the breast contour estimate gc chosen for the breast-centering assessment, at each point X along this test contour, the minimum bistatic distance $r_{gc}\left(x\right)$ between this point and any pair of RF sensors (among the reduced set of pairs preselected for use with this estimation module) that can “see” that point, is computed.

In order to assess the centering quality of the estimated contour, an ideally centered reference contour is derived by translating the estimated contour by a varying amount $x_{T}$ around the 2D region of interest until it yields the largest value of $\oint_{gc_{T}}r_{gc_{T}}\left(gc_{T}\left(s\right)\right)ds$, where $gc_{T}=gc+x_{T}$ is the translated contour and $r_{gc_{T}}\left(x\right)$ denotes the minimum bistatic distance to a point x on this translated contour. For this ideal centered reference contour, the minimum bistatic distances, associated to each point x of this curve, denoted by $r_{c}\left(x\right)$, are similarly calculated as for the estimated contour.

For brevity, the notation x is used in the sequel of this section to refer to a given point along the estimated breast contour, and also to refer to the corresponding point $x+x_{T}$ on the ideally centered reference contour.

The centering assessment is then performed by comparing the bistatic ratio:

(18) $$b_{r}:=\underset{x\in gc}{\max}\left(\frac{\left|r_{c}\left(x\right)-r_{gc}\left(x\right)\right|}{r_{c}\left(x\right)}\right)$$

to the threshold value $B_{r}\cdot p_{dist\_thresh}$ where $B_{r}$ is the largest bistatic ratio of any possible translation of the estimated contour, and $p_{dist\_thresh}$ is a user-defined parameter, which is by default set to 0.85 for the system validation test.

If $b_{r}$ exceeds $B_{r}\cdot p_{dist\_thresh}$, then the estimated breast contour is marked as remarkably off-centered, and the breast centering confidence level P is set to a minimal value that is preset via the parameter $perc_{at\_\max\_dis\tan ce}$. Otherwise, the ratio $b_{r}/(B_{r}\cdot p_{dist\_thresh})$ is used to compute the breast centering confidence level, as defined in Equation (19):

(19) $$p=\{\begin{array}{ll} 100\cdot perc_{at\_\max\_dis\tan ce}, & if\;b_{r}>B_{r}\cdot p_{dist\_thresh}\\ 100-100(1-perc_{at\_\max\_\mathrm{distance}})\frac{b_{r}}{B_{r}\cdot p_{dist\_thresh}}, & otherwise \end{array}$$

To this extent, the centering assessment will have a confidence level percentage ranging between a maximum of 100 (if the test contour is coincident with the ideal centered contour) and a minimum of $100\cdot perc_{at\_\max\_\mathrm{distance}}$. For the on-site validation tests, the minimal value 50% has been used for all of the centering assessment tests.

**Figure 13:**
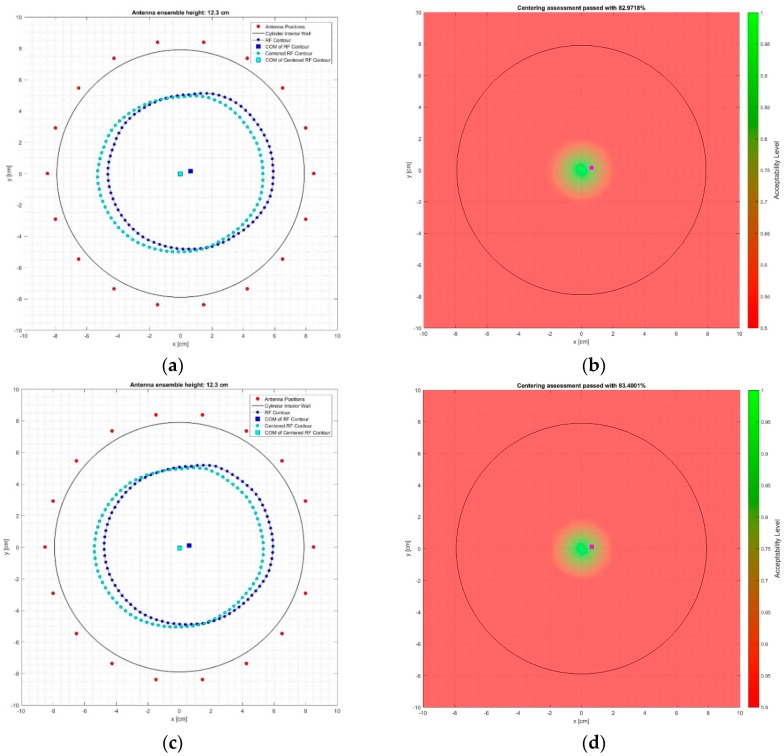
Breast rotational position #1: (**a**) Test Date 1, estimated outer breast surface at a given coronal slice; (**b**) Test Date 1, breast centering assessment map; (**c**) Test Date 2, estimated outer breast surface at a given coronal slice; (**d**) Test Date 2, breast centering assessment map.

In Figure 13a,c the breast surface contour estimate chosen for the breast centering assessment (depicted in blue) and the associated ideally centered contour (depicted in cyan) are shown for the tests at the breast rotational position #1, on Test Date 1 and Test Date 2, correspondingly. The red dots depict the location of the sensors, while the black circle represents the inner wall of the transition liquid container.

The associated spatial map of breast-centering assessment is shown in Figure 13b,d for the two test dates of the breast rotational position #1, correspondingly.

In either figure, the purple square represents the location of the center of mass of the breast surface contour estimate that was used for the breast-centering assessment; the resulting breast-centering confidence level is marked on the title of each figure. The black circle depicts the inner wall of the transition liquid container.

**Figure 14:**
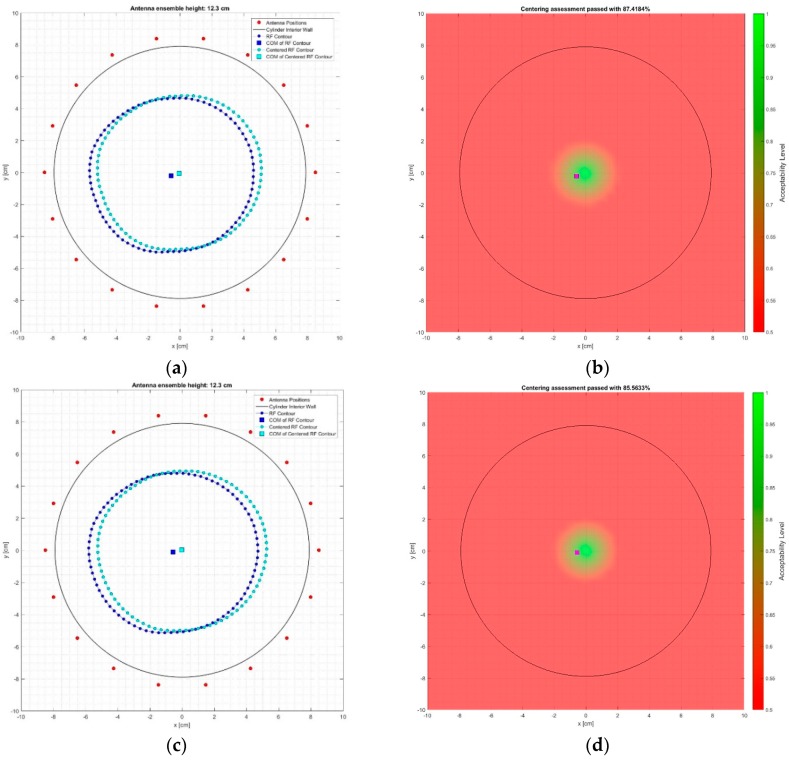
Breast rotational position #2: (**a**) Test Date 1, estimated outer breast surface at a given coronal slice; (**b**) Test Date 1, breast centering assessment map; (**c**) Test Date 2, estimated outer breast surface at a given coronal slice; (**d**) Test Date 2, breast centering assessment map.

The results for the breast rotational position #2 are given in Figure 14a,b for the data recorded on Test Date 1, and in Figure 14c,d for Test Date 2. All of the notations are consistent with the definitions provided earlier as explanation to Figure 13.

The breast-centering confidence levels, as computed for all four test cases, are comparatively presented in Table 3.

It is shown that the natural off-centering of the breast phantom at the selected coronal slice is repeatedly identified with a fair level of accuracy, associated with centering confidence levels varying between 83–87.4%. It is worth noting that a slight offset is repeatedly identified between the estimates for the two distinct rotational positions of the breast phantom. This is an indication of a slight non-symmetry in the reconstructed geometry, introduced either by the sensor network itself, or possibly due to a non-homogeneous thermal distribution in the interior of the examination table.

#### 2.6.5. Image Quality Assessment (QA) Metrics for System Performance Acceptance

The microwave breast imaging system evaluation and acceptance is performed based on a series of quality metrics which are computed on multistatic radar images of the breast phantoms, as defined in Equations (13) and (14).

- QA Metric 1: Focusing Metric (FM) evaluated on the composite image formed at single vertical position of the sensor network (as per Equation (13)), in front of the tumor:

The FM is evaluated on a series of images, parameterized by the assumed percentage of fibroglandular tissue $pc_{fib}$ in the breast.

◌ Acceptance Criterion (AC) #1: The optimal $pc_{fib}$ value, for which the focusing measure is maximized, should remain constant at every repetition of the controlled imaging test, and for every rotational position of the breast phantom (testing with $pc_{fib}$ intervals equal to 5%).
◌ AC#2: the value of the focusing measure, for the optimal $pc_{fib}$, should exceed a preset threshold value $thr_{QA\_1}$.
  - QA Metric 2: Intensity of the TR-MUSIC pseudospectrum at the tumor location (Im_max_), evaluated on the composite image formed at a single vertical position of the sensor network (as per Equation (13)), in front of the tumor:

The Im_max_ is evaluated for a series of images parameterized by the assumed percentage of fibroglandular tissue $pc_{fib}$ in the breast:

◌ AC#3: The optimal $pc_{fib}$ value for which Im_max_ is maximized should remain constant at every repetition of the controlled imaging test, and for every rotational position of the breast phantom (testing with $pc_{fib}$ intervals equal to 5%).
◌ AC#4: The value of Im_max_, for the optimal $pc_{fib}$ value, should exceed a preset threshold value $thr_{QA\_2}$.
◌ AC#5: The two patterns FM($pc_{fib}$) and Im_max_($pc_{fib}$) should be consistent with each other, meaning that maximization and identical slope(s) are observed for the same $pc_{fib}$ values on both patterns.
  - QA Metric 3: Variation of the maximal achievable focusing FM over the height, evaluated for images formed using various vertical scan positions of the sensor network (as per Equation (14)):
◌ AC#6: The maximal FM should be observed at the same height: the one closer to the tumor, at every repetition of the controlled imaging test, and for every rotational position of the breast phantom.
◌ AC#7: The contrast between the maximal FM and the FM achievable at all of the other heights should exceed a given threshold $thr_{QA\_3}$.
  - QA Metric 4: Ratio between the average image intensity at the exterior of the breast and the Im_max_ in the interior of the breast: Evaluation on the composite image formed using data from multiple vertical scan positions of the sensor network (as per Equation (14)):

The multi-height image is formed via concatenation of the single-height images with $pc_{fib}$ automatically selected to allow optimal image focusing independently per height.

◌ AC#8: The ratio should not exceed a preset upper-limit value $UL_{QA\_4}$.

## 3. Results

In this section, indicative results are presented from the test campaign that has been recently carried out for the site acceptance of the Wavelia microwave breast imaging system after its installation at the Galway University Hospital for a pilot first-in-human clinical test [63]. The series of image quality assessment (QA) metrics, as defined in Section 2.6.5, have been evaluated on four scans of a realistically complex breast phantom, as detailed in Section 2.6.3.

### 3.1. QA Metrics 1 and 2: Images Formed at Single Vertical Position of the Sensor Network

#### 3.1.1. Breast Rotational Position #1

**Figure 15:**
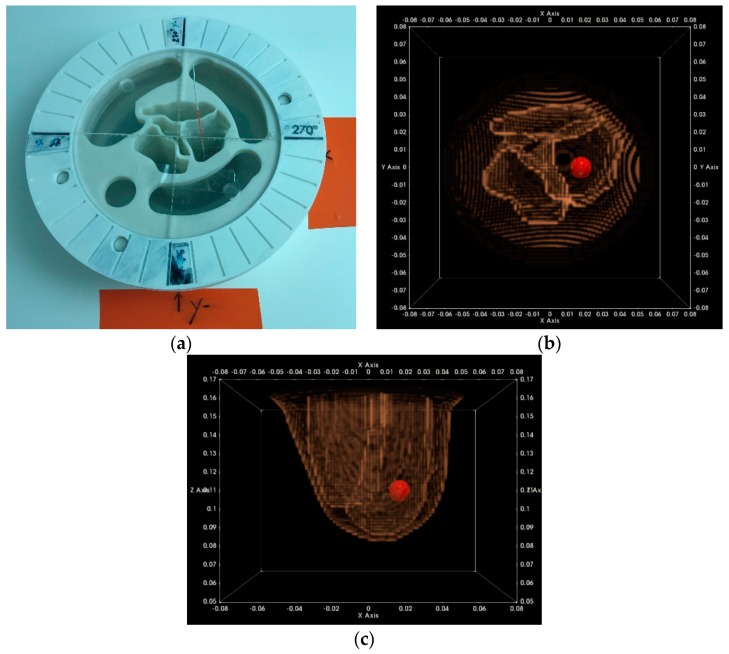
Experimental setup for the breast rotational position #1: (**a**) Photo—Top view of the breast phantom, installed on the examination table; (**b**) Schematic definition of the tumor location (red sphere with a 14-mm diameter, equal to the average diameter of the microlobulated tumor phantom), in the fibroglandular tissue of the breast (the outer surface of both the fibroglandular mold and the outer breast surface mold are depicted with orange color)—Top View; (**c**) Schematic definition of the tumor location in the fibroglandular tissue of the breast—Side View.

In Figure 15, the experimental setup for the tests at the rotational position #1 of the breast phantom is illustrated. For these tests, the microlobulated tumor of average size (14 mm) has been immersed in the fibroglandular tissue-mimicking liquid, at the location (*x*, *y*, *z*) = (20, 0, 110) mm (=center of the tumorous lesion).

The test has been repeated on two distinct dates. Imaging results from the two identical tests are presented and compared in this section.

**Figure 16:**
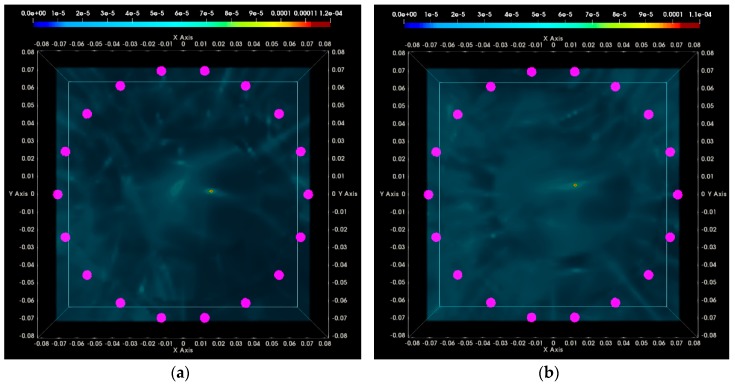
Breast rotational position #1—image formed at a single vertical position of the sensor network, in front of the tumor; top XY view of the full imaging domain (the antennae center positions are depicted with purple dots): (**a**) Test Date 1; (**b**) Test Date 2.

In Figure 16, the composite TR-MUSIC pseudospectra, as formed using Equation (13) and data from a single vertical scan position of the sensor network, are depicted for the two data snapshots recorded on two different dates. The full imaging domain, both in the interior and the exterior of the breast phantom, is evaluated. The objective of such a visualization is to highlight the absence of any significant artifact radar echoes at the exterior of the breast, in the case of both measurements.

These composite images have been formed with the integration of monochromatic (single-frequency) TR-MUSIC pseudospectra, computed as per Equations (9)–(13), using:

▪ a given number Nfreq of frequency points, uniformly spanning the working frequency band,
▪ a given number Nsec of sectors of antenna sub-arrays spanning the full 360° azimuth domain around the breast.

The images that have been formed under the assumption $pc_{fib}$ that resulted in maximized focusing are here depicted. The optimal $pc_{fib}$ has been automatically selected with the method that has been defined in Section 2.4.5.

The applied data processing chain is meant to result, ideally, in the formation of very spiked images indicating the probability of the target presence on each pixel of the imaging domain. The unambiguously detected and accurately localized targets are expected to be associated with constellations of very small bright spots, highlighting the target position in an overall dark spatial map. In Figure 16a,b, a clear and pronounced peak of the TR-MUSIC pseudospectrum is visible on both images in the vicinity of the ground truth location of the tumor.

**Figure 17:**
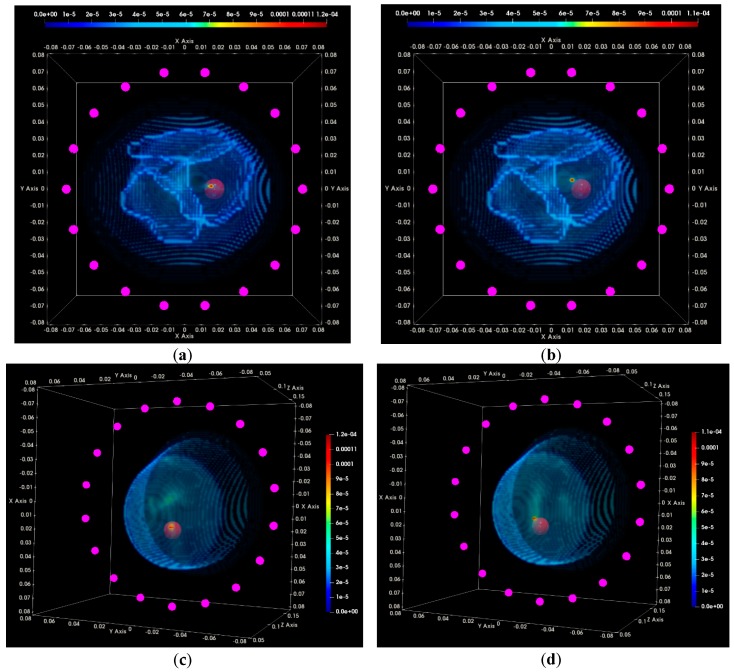
Breast rotational position #1—image in the interior of the breast only, formed at a single vertical position of the sensor network, in front of the tumor: (**a**) Test Date 1, top XY view, superposition of the fibroglandular and outer breast surface molds (blue color), red sphere indicating the tumor location; (**b**) Test Date 2, top XY view, superposition of the fibroglandular and outer breast surface molds (blue color), red sphere indicating the tumor location; (**c**) Test Date 1, 3D view, superposition of the outer breast surface mold (blue color), red sphere indicating the tumor location; (**d**) Test Date 2, 3D view, superposition of the outer breast surface mold (blue color), red sphere indicating the tumor location.

It is noticeable that the intensity of the TR-MUSIC pseudospectrum is slightly higher on the first Test Date 1, as compared to Test Date 2. In addition, two secondary radar echoes (of significantly lower intensity compared to the dominant echo, which is clearly attributed to the tumor) are present on the image of Test Date 1. These secondary echoes can be attributed to a “cavity” of adipose tissue that is formed in between the three compartments of the mold filled with fibroglandular tissue-mimicking liquid in the breast phantom. This adipose ‘cavity’, which has significant negative dielectric contrast with respect to the surrounding fibroglandular tissue, is visible in Figure 17a, and can be spatially correlated with the secondary radar echoes seen in Figure 16a.

In Figure 17a,b, the same images as Figure 16a,b are shown, but after having filtered out the parts corresponding to the exterior of the breast phantom. The breast external contour has been a priori extracted from the data as defined in Section 2.4.2, and is used here for spatial filtering in order for the image to be easier interpretable from a physical point of view. The borders of the fibroglandular tissue-mimicking molds, which are a priori known, and a red sphere with diameter equal to the average size of the microlobulated tumor, have also been superimposed on the images in Figure 17a,b. The objective of this second visualization is a straightforward linking of the bright spots on the images of Figure 16a,b and the experimental setup.

In Figure 17c,d, an alternative viewpoint is provided for the same images of the breast interior. The selected viewpoint would correspond to a front-side view of the breast, while the patient is in the standing position. The borders of the fibroglandular tissue mimicking molds have not been superimposed with the images in this third visualization.

Clean images that can be clearly associated with unambiguous detection of the tumor have been retrieved on both tests of the rotational position 1 of the breast phantom.

**Figure 18:**
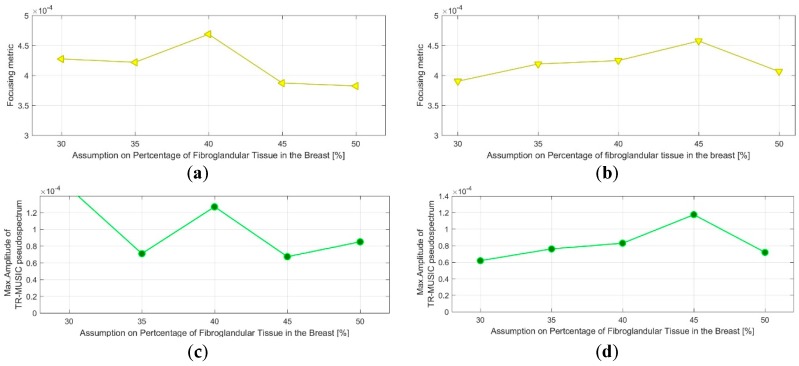
Breast rotational position #1: (**a**) Test Date 1, image quality assessment (QA) metric 1; (**b**) Test Date 2, image QA metric 1; (**c**) Test Date 1, image QA metric 2; (**d**) Test Date 2, image QA metric 2.

The evaluation of the QA metric 1 is shown in Figure 18a,b for the two images, formed on Test Date 1 and Test Date 2 correspondingly. It can be observed that maximal focusing is achieved for $pc_{fib}$ = 40% on Test Date 1, while on Test Date 2, the optimal $pc_{fib}$ value is 45%.

The acceptance test (AC) #1 would strictly fail in such a case. However, given the proximity of the ‘average’ breast tissue dielectric properties that are associated with the two $pc_{fib}$ values, as depicted in Figure 8, and also considering the constrained and yet non-optimized stability and robustness of both the imaging system and the transition liquid itself against slight variations in the nominal environmental operating conditions (e.g., slight temperature variations), such a variation in the optimal $pc_{fib}$ value, in terms of focusing, is still considered acceptable for the on-site validation tests of the actual version of the imaging system prototype.

The threshold value for the optimal focusing metric (FM) per image has been set to $thr_{QA\_1}=0.0004$. This is valid for the specific experimental setup, which has been reproduced both at factory and after system installation on-site. This is the threshold value that is used with acceptance test #2 all along the on-site validation of the imaging system. Both tests at the rotational position 1 of the breast are thus validated in terms of AC #2.

The evaluation of the QA metric 2 is shown in Figure 18c,d for the two images, formed on Test Date 1 and Test Date 2, correspondingly. It can be observed that the maximal intensity Im_max_ of the TR-MUSIC pseudospectra is maximized for the same $pc_{fib}$ values as the FM. Concerning acceptance test #3, the same considerations hold as for AC#1. In terms of acceptance test #4, the threshold value for the image intensity at the target (tumor) position has been set to $thr_{QA\_2}=0.0001$, while performing tests with the same experimental setup as at the factory. This is the threshold value that is used with the AC #4 all along the on-site validation of the imaging system. Both tests at the rotational position 1 of the breast are thus validated in terms of AC #4. Finally, the two patterns FM ($pc_{fib}$) and Im_max_ ($pc_{fib}$) remain consistent between each other, as far as the dependence on $pc_{fib}$ is concerned, with the exception of the outlier point: Im_max_ ($pc_{fib}$), $pc_{fib}$ = 30%. Acceptance test #5 is validated in such a case of similarity between the two patterns at the given prototype state of the imaging system.

#### 3.1.2. Breast Rotational Position #2

**Figure 19:**
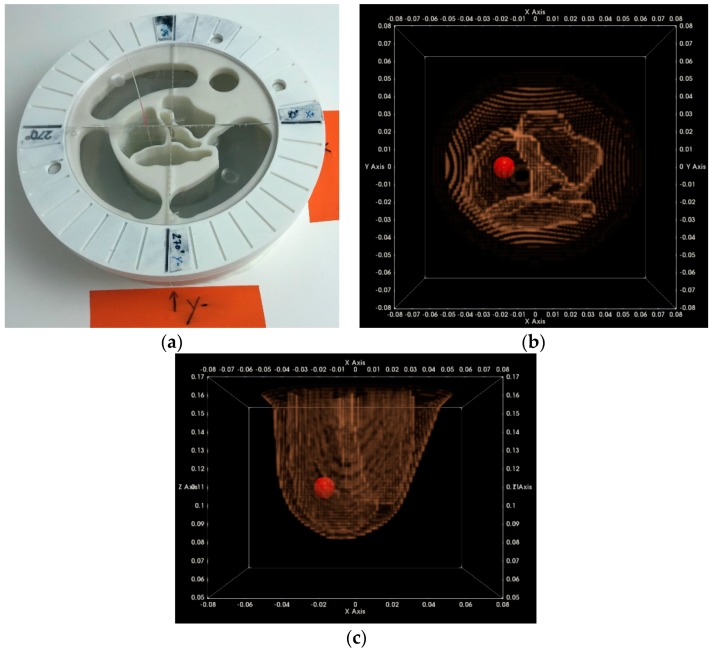
Experimental setup for the breast rotational position #2: (**a**) Photo—Top view of the breast phantom, installed on the examination table; (**b**) Schematic definition of the tumor location (red sphere of diameter 14 mm, equal to the average diameter of the microlobulated tumor phantom) in the fibroglandular tissue of the breast (the outer surface of both the fibroglandular mold and the outer breast surface mold are depicted with orange color)—Top View; (**c**) Schematic definition of the tumor location in the fibroglandular tissue of the breast—Side View.

In this section, the same QA metrics 1 and 2 are evaluated for the two imaging tests that have been performed at the rotational position 2 of the same breast phantom on two distinct dates: Test Date 1 and Test Date 2. The breast phantom is rotated by 180°, with respect to the two first tests, which have been thoroughly evaluated and validated in terms of QA 1 and QA 2 in the previous section. In Figure 19, the experimental setup for the tests at the rotational position #2 of the breast phantom is illustrated.

For these tests, a microlobulated tumor of average size (14 mm) has been immersed in the fibroglandular tissue-mimicking liquid at the location (*x*, *y*, *z*) = (−20, 0, 110) mm (=center of the tumorous lesion).

**Figure 20:**
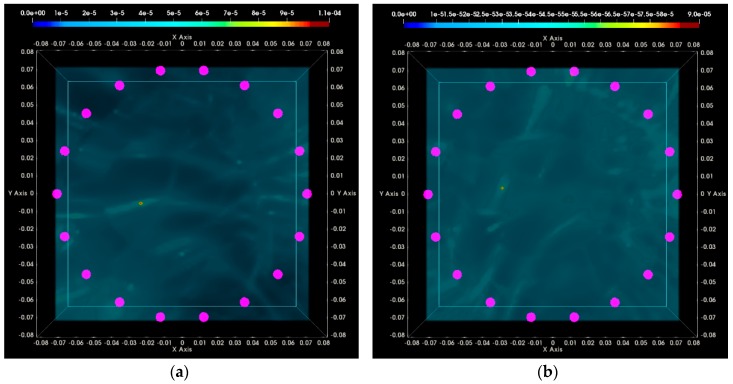
Breast rotational position #2—image formed at a single vertical position of the sensor network, in front of the tumor, top XY view of the full imaging domain (the antennae center positions are depicted with purple spots): (**a**) Test Date 1; (**b**) Test Date 2.

In Figure 20, the composite TR-MUSIC pseudospectra, as formed using the Equation (13), and data from a single vertical scan position of the sensor network are depicted for the two data snapshots recorded on two different dates.

**Figure 21:**
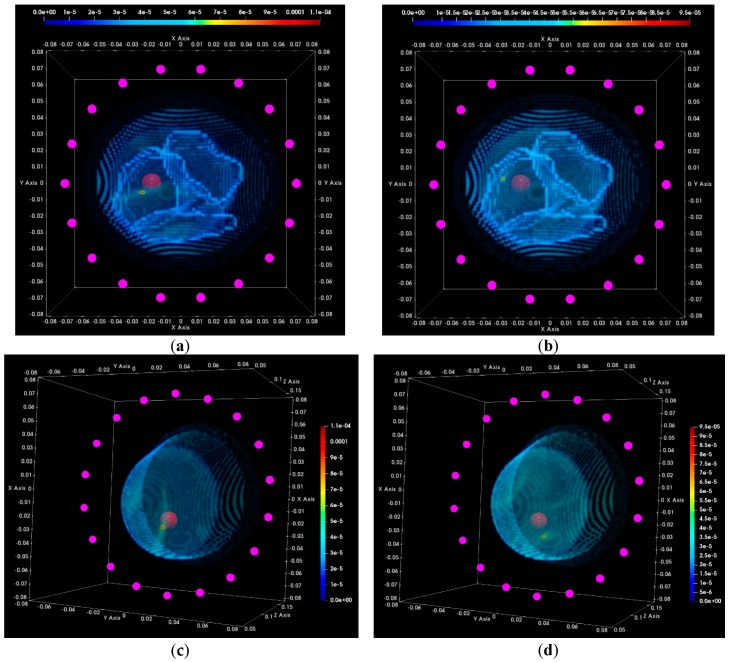
Breast rotational position #2—image in the interior of the breast only, formed at a single vertical position of the sensor network, in front of the tumor: (**a**) Test Date 1, top XY view, superposition of the fibroglandular and outer breast surface molds (blue color), red sphere indicating the tumor location; (**b**) Test Date 2, top XY view, superposition of the fibroglandular and outer breast surface molds (blue color), red sphere indicating the tumor location; (**c**) Test Date 1, 3D view, superposition of the outer breast surface mold (blue color), red sphere indicating the tumor location; (**d**) Test Date 2, 3D view, superposition of the outer breast surface mold (blue color), red sphere indicating the tumor location.

These images have been formed in exactly the same way, as detailed in Section 3.1.1 for the images in Figure 16. A clear and pronounced peak of the TR-MUSIC pseudospectrum is visible on both images in the vicinity of the ground truth location of the tumor. However, when comparing these images with the ones in Figure 16, it is noticeable that the maximal intensity of the TR-MUSIC pseudospectrum in Figure 20b is lower than the maximal intensity in the three other images. The dominant peak that is unambiguously associated with the tumor multistatic radar echo is also slightly misplaced with respect to the ground truth location of the tumor. The observed shift can be better seen in Figure 21b. The four images in Figure 21 have been formed in exactly the same way as the corresponding images in Figure 17 in Section 3.1.1.

**Figure 22:**
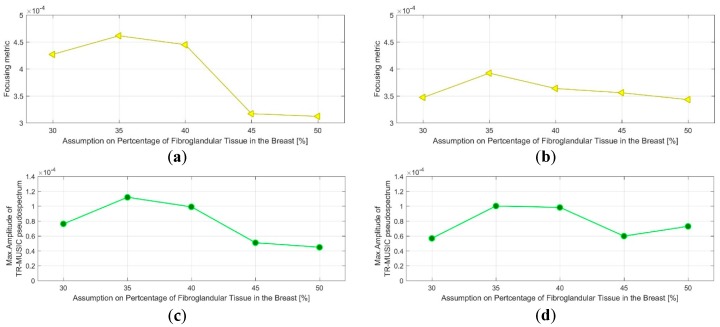
Breast rotational position #2: (**a**) Test Date 1, image QA metric 1; (**b**) Test Date 2, image QA metric 1; (**c**) Test Date 1, image QA metric 2; (**d**) Test Date 2, image QA metric 2.

Clean images that can be clearly associated with unambiguous detection of the tumor have been retrieved from both tests at rotational position 2 of the breast phantom. The imaging performance is slightly degraded on Test Date 2; however, such a level of degradation lies within the limits of acceptable variability in the system performance at this stage of development. All four datasets presented in the article are thus examples of test data that have served the on-site validation of the imaging system. The quantified evaluation of the system performance, in terms of the QA metrics 1 and 2, is shown in Figure 22 for the two tests at rotational position 2 of the breast phantom.

The result representation in Figure 22 is identical to the one in Figure 18 for the two tests at rotational position 1 of the breast phantom, which has been detailed in Section 3.1.1.

It can be observed in Figure 22a,b that maximal focusing is achieved for $pc_{fib}$ = 35% on both test dates. The optimal $pc_{fib}$ value remains constant between the two test dates, as required by acceptance test #1; however, this value is lower than the optimal value identified for rotational position 1 of the breast phantom. This phenomenon of slightly shifted optimal $pc_{fib}$, depending on the orientation of the breast phantom with respect to the sensor network, has been consistently observed on more validation test datasets of the imaging system, and could be attributed to the slight inhomogeneity in the temperature spatial distribution in the interior of the device, at its actual version. This is accepted as such, and validated for the clinical pilot testing of the system; a thermoregulation of the device interior is planned to be put in place when upgrading the device design in the future, such that this type of inhomogeneity can be avoided. The ‘average’ breast tissue dielectric properties that are associated with each $pc_{fib}$ value are defined in Figure 8.

Considering the threshold value $thr_{QA\_1}=0.0004$ for the optimal focusing metric (FM) per image, as defined in Section 3.1.1, acceptance test #2 is clearly validated on Test Date 1, but it is hardly reached on Test Date 2, as can be observed in Figure 22a,b.

The evaluation of the QA metric 2 is shown in Figure 22c,d. It is shown that the maximal intensity Im_max_ of the TR-MUSIC pseudospectra is maximized for the same $pc_{fib}$ values as the FM, such that AC #3 is validated on both test dates. Given the threshold value for the image intensity at the target (tumor) position, $thr_{QA\_2}=0.0001$, as specified in Section 3.1.1, AC #4 is clearly validated on Test Date 1 and just met on the Test Date 2.

The two patterns: FM ($pc_{fib}$) and Im_max_ ($pc_{fib}$) remain consistent between each other, as far as the dependence on $pc_{fib}$ is concerned; acceptance test #5 is validated on both test dates.

### 3.2. QA Metrics 3 and 4: Images Formed at Multiple Vertical Positions of the Sensor Network

**Figure 23:**
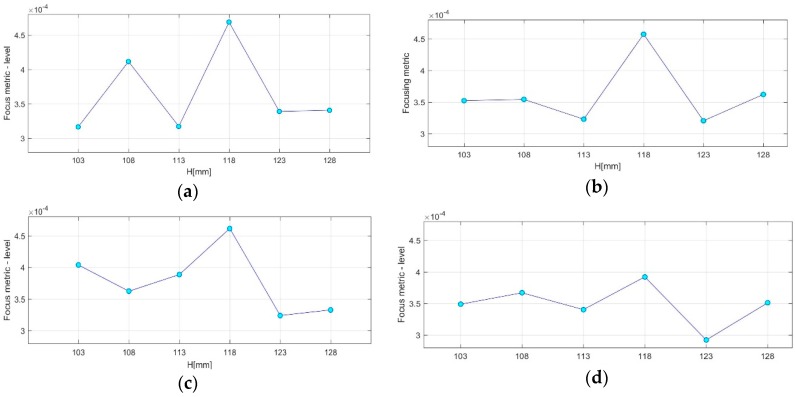
Imaging focusing analysis on 3D multi-H data: (**a**) Rotational position #1—Test Date 1; (**b**) Rotational position #1—Test Date 2; (**c**) Rotational position #2—Test Date 1; (**d**) Rotational position #2—Test Date 2.

In Figure 23, the maximal focusing metric (FM), as extracted from Figure 18a,b and Figure 22a,b for the four test datasets at single H = 118 mm (sensor network in front of the tumor), is plotted as evaluated on images that have been formed using six different vertical scan positions of the sensor network (vertical sampling rate = 5 mm). The result, which is QA metric 3 as defined in Section 2.6.5, is plotted in Figure 23a,b for the breast rotational position 1, Test Date 1, and Test Date 2, correspondingly. In Figure 23c,d, QA metric 3 is plotted for the breast rotational position 2, Test Date 1, and Test Date 2, accordingly. The maximal FM is observed at the same height, H = 118 mm, for both rotational positions of the breast phantom, and for both repetitions of either of the two controlled imaging tests. AC #6 is validated based on the results presented for the four test datasets, as shown in Figure 23.

Ideally, an overall contrast between the maximal FM (at H = 118 mm, coronal slice of the breast on which the tumor is better ‘seen’ by the sensor network) and the FM that is achievable at any other coronal breast slice should exceed a given threshold $thr_{QA\_3}=1.2$, as is the case in Figure 23b for breast rotational position 1 on Test Date 2. While such a case represents the goal in terms of unambiguous retrieval of the tumor echo along the vertical scan of the heterogeneous breast, AC #7 is validated also in the case of Figure 23a,c, where the contrast in terms of FM exceeds the value $thr_{QA\_3}=1.1$. In the case of Figure 23d, the computed contrast is 1.08. It has been concluded in the course of the on-site validation of the imaging system that the three first test datasets are validated in terms of AC #7, while the fourth test dataset hardly meets the set threshold value. It is interesting to notice that the breast rotational position 2—Test Date 2 scan is the only one that has been marked as invalid (or potentially critically valid) by the total of three quantitative evaluation tests: AC#2, AC#4, and AC#7.

**Figure 24:**
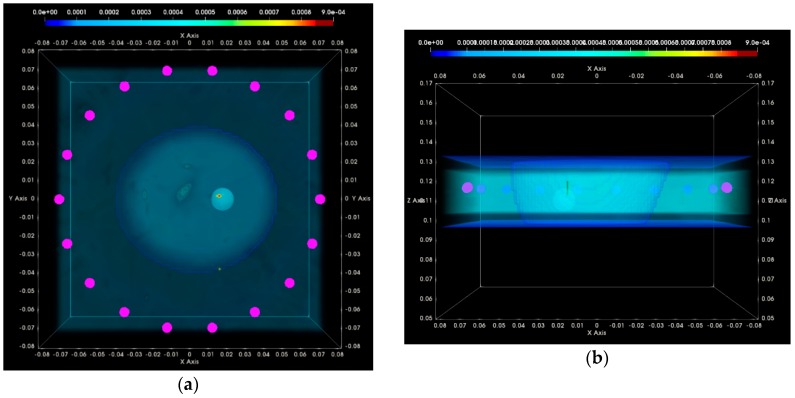
Breast rotational position #1—Test Date 1—image formed using six vertical positions of the sensor network in the vicinity of the tumor—image in the interior of the breast superimposed with the image of the full imaging domain: (**a**) top XY view; (**b**) side XZ view.

In Figure 24, a top and side view of the composite image formed using the data from the six vertical scan positions of the sensor network (as per Equation (14)) are shown for breast rotation position 1 on Test Date 1. The ground truth location of the tumor phantom is illustrated with a spherical inclusion with a diameter of 14 mm, which is equal to the average size of the microlobulated tumor that is superimposed on the images. This type of multi-height composite image has been formed via concatenation of the single-height images with automatically selected $pc_{fib}$, to allow optimal image focusing independently per height, as explained in Section 2.4.5. The sensor positions, as mapped on the inner wall of the container filled with transition liquid, are illustrated with the purple dots that are overlaid on the images. Overlapping zones exist in the 3D imaging domain among the elementary images formed from data at a single vertical scan position of the sensor network. Intensity normalization operations are also involved in the concatenation of the elementary images for formation of the composite multi-height image; this is the reason why the scaling of the intensity is different for the single-height (formed as per Equation (13)) and the multi-height images (formed as per Equation (14)). The difference in scaling depends on the number of integrated vertical scan positions and the amount of overlap among the elementary images. These parameters are not detailed any further in this paper.

**Figure 25:**
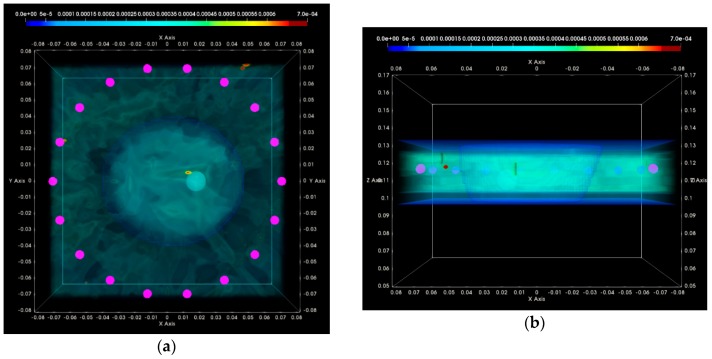
Breast rotational position #1—Test Date 2—image formed using six vertical positions of the sensor network, in the vicinity of the tumor—image in the interior of the breast superimposed with the image of the full imaging domain: (**a**) top XY view; (**b**) side XZ view.

In Figure 25, a top and side view of the composite image formed using the data from the six vertical scan positions of the sensor network (as per Equation (14)) are shown for breast rotation position 1 on Test Date 2.

By comparing the imaging results in Figure 24 and Figure 25, it is clear that while unambiguous detectability of the tumor in the breast interior is assured all along the six vertical scan positions, the maximal intensity of the composite TR-MUSIC pseudospectrum in the breast (tumor constellation of echoes) is lower in Figure 25 as compared to Figure 24. Few spots of unfiltered clutter/interference close to the sensor network are also visible in the images in Figure 25. It is well seen on the side view in Figure 25b that the unfiltered interferers appear a bit higher than the tumor (=closer to the examination table).

**Figure 26:**
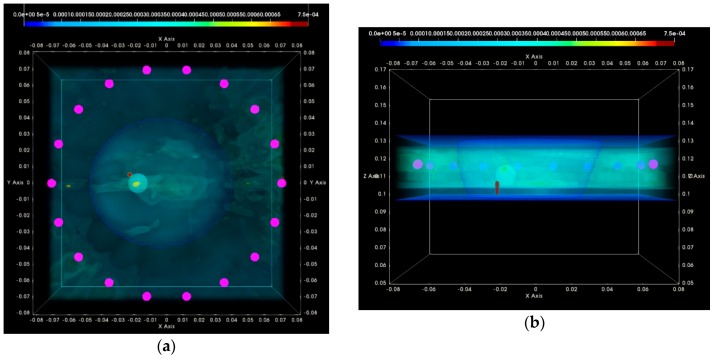
Breast rotational position #2—Test Date 1—image formed using six vertical positions of the sensor network, in the vicinity of the tumor—image in the interior of the breast superimposed with the image of the full imaging domain: (**a**) top XY view; (**b**) side XZ view.

**Figure 27:**
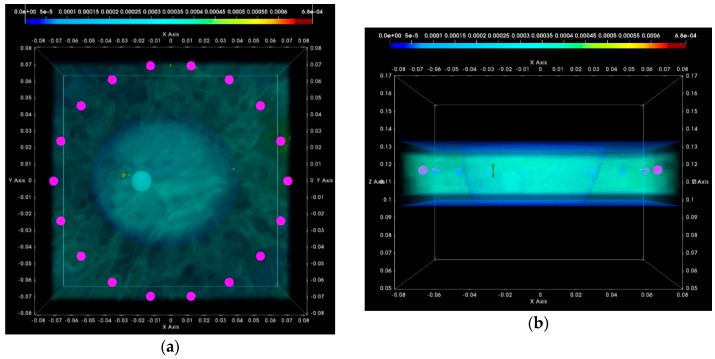
Breast rotational position #2—Test Date 1—image formed using six vertical positions of the sensor network, in the vicinity of the tumor—image in the interior of the breast superimposed with the image of the full imaging domain: (**a**) top XY view; (**b**) side XZ view.

In Figure 26 and Figure 27, a top and side view of the composite images formed using the data from the same six vertical scan positions of the sensor network (as per Equation (14)) are shown for breast rotation position 2, on Test Date 1 and Test Date 2, correspondingly. The image intensity associated with the constellation of tumor radar echoes is a bit lower on both test dates, as compared to the images in Figure 24. The constellation of more than a single peaked spot is associated with the tumor on the TR-MUSIC pseudospectra of Figure 26. This is acceptable, given the size and irregular shape of the target, as seen in Figure 5.

In both Figure 26 and Figure 27, a slightly higher level of overall intensity in the exterior of the breast phantom (level of residual interferer echoes) is observed, as compared to the images in Figure 23 and Figure 24. This is an indicator of the slightly degraded imaging performance of the system in the case of breast rotational position 2 on both test dates. This is quantifiable by means of QA metric 4, i.e., the ratio between the average image intensity in the non-focused image in the exterior of the breast versus the maximal image intensity in the focused image in the interior of the breast (clearly associated with the tumor radar echo on all the presented images). The values of QA metric 4 are given in Table 4 for all four composite images in Figure 24, Figure 25, Figure 26 and Figure 27. Ideally, QA metric 4 should not exceed the upper limit value $UL_{QA\_4}=10\%$ for on-site acceptance of a test scan using the specific controlled imaging scenario. The scans of both breast rotational positions on Test Date 2 (this is not the same date for both scans) are thus at the limit of being marked as incompatible in terms of AC #8.

## 4. Discussion and Conclusions

In Table 5, a summary of the acceptance test results is reported for the four breast phantom scans, which have been thoroughly analyzed in Section 3.

Site acceptance of the imaging system is suggested, if more than N_test_/2 valid tests are consistently reported, at every scan repetition, during a one-week test validation period (N_test_ = 8 is the total number of acceptance tests performed and evaluated after each scan, as defined in Section 2.6.5).

This summarized result presentation makes clear the degradation that has been observed for the scan at breast rotational position #2, on Test Date 2, when compared to the other three scans, in terms of the defined QA metrics. This is indicative of the expected and acceptable level of variability in the performance of the imaging system prototype.

At this stage of system development, and toward pilot clinical testing, all the “critically valid” AC test results in Table 5 have been considered acceptable. On-site acceptance of the imaging system is validated with such results, provided that such a performance is consistently achieved along the total duration of the one-week validation period.

In the case of the breast phantom defined in Section 2.3 and used for the validation tests of the system, even if a single tumor model is inserted in the breast phantom under test, the complex geometry of the plastic molds, filled with either adipose or fibroglandular tissue-mimicking liquid, may result in unfiltered radar echoes originating from the corners on the mold surface, which may be erroneously seen as “scattering objects of interest”. This complexity renders the test scenario, which is used for system validation, particularly challenging (from a radar point of view), and does not necessarily correspond to a physical complexity that is expected to be found in the real breast; less discontinuous transitions and a less structured multi-layered configuration is naturally expected to be found in the real breast, but cannot be easily reproduced in a phantom.

On the other hand, it is clear that any perturbation that may be introduced in the microwave breast scan due to either an intentional motion of the patient or unintentional ‘micromotions’ of living body cells during the scan, has not been considered so far, and its impact will be investigated based on clinical data only. Interference due to blood flow in the breast, or due to the interface between the examination table and the patient’s chest wall have not been investigated either. The inherent interpatient anatomical variability and its impact on the pre-processing modules for sensor coupling and breast skin echo suppression will also need to be carefully investigated during the pilot clinical test. Finally, the interpatient variability in terms of: normal and cancerous breast tissue dielectric properties and associated contrasts, breast density, and skin texture depending on age, are all examples of physical phenomena that have not been modeled by the phantoms used for the system design and validation.

Given the above considerations on potential sources of mismodeling of the breast with the available phantoms, it is inevitable that some adjustments of both the hardware and software modules of the system architecture may need to be performed as a conclusion of the planned pilot clinical testing. Such adjustments may be required such that the intended imaging performance, as validated with the indicative results presented in this paper, is assured and validated when processing the clinical data from patient breast scans as well.

## Figures and Tables

**Table 1:** Breast Phantom Metrology, Based on the Optical Scan.

Measures	Breast Phantom #1		Breast Phantom #2	
	Site	Factory	Site	Factory
Breast Volume (mL)	698	696	1099	1097
Breast Vertical Extent (mm)	85	84	108	108

**Table 2:** Temperature Measurement Conditions: Breast Phantom Validation Test.

Transition Liquid		Fibroglandular Tissue	
Before the Scan	After the Scan	Before the Scan	After the Scan
22.7 °C–23.3 °C	23.0 °C–24.1 °C	20.6 °C	21.2 °C

**Table 3:** Confidence Level for Breast Centering.

Breast	Test Date 1	Test Date 2
Rotational position #1	82.97%	83.40%
Rotational position #2	87.42%	85.86%

**Table 4:** QA Metric 4, Evaluated for the Four Analyzed Test Datasets.

Breast	Test Date 1	Test Date 2
Rotational position #1	7.41%	10.38%
Rotational position #2	4.68%	11.42%

**Table 5:** Summary of Acceptance Test Results.

Acceptance Test (AC)	Rotational Position #1		Rotational Position #2	
	Test Date 1	Test Date 2	Test Date 1	Test Date 2
#1	-	-	-	-
#2	+	+	+	×
#3	-	-	-	-
#4	+	+	+	-
#5	-	+	+	+
#6	+	+	+	+
#7	-	+	-	×
#8	+	-	+	-

+, Valid; -, Critically Valid; ×, Invalid.
